# Intracrine VEGF Signaling Is Required for Adult Hippocampal Neural Stem Cell Maintenance and Vascular Proximity

**DOI:** 10.1007/s12035-025-04861-1

**Published:** 2025-03-25

**Authors:** Tyler J. Dause, Robert Osap, Akela A. Kuwahara, Jiyeon K. Denninger, Elizabeth D. Kirby

**Affiliations:** 1 Department of Psychology, College of Arts and Sciences, The Ohio State University, Columbus, OH USA; 2 Department of Cell and Tissue Biology, University of California San Francisco, San Francisco, CA USA; 3 Chronic Brain Injury Program, The Ohio State University, Columbus, OH USA

**Keywords:** Dentate gyrus, Neural stem cells, Vascular endothelial growth factor

## Abstract

Adult neural stem cells (NSCs) in the mammalian dentate gyrus (DG) of the hippocampus rely on multiple signals for their preservation throughout the lifespan. While several studies have suggested that vascular endothelial growth factor (VEGF), in particular VEGF synthesized by NSCs themselves, is critical for NSC maintenance and adult neurogenesis, conflicting studies have left it uncertain how VEGF signals to NSCs. Here, we identified a VEGF-VEGFR2 intracrine signaling mechanism within adult DG NSCs that prevents NSC exhaustion and supports their proximity to local blood vessels. Using cell culture assays, we show that while intracellular VEGF stimulated receptor signaling cascades, extracellular VEGF did not. We found that this primary reliance on intracellular VEGF receptor signaling was most likely due to sheddase-mediated cleavage of extracellular VEGFR2 ligand binding domains, as phospho-signaling in response to extracellular VEGF could be restored using sheddase inhibitors. Using cultured adult DG NSCs and intact mice, we further show that NSC-VEGF loss caused cell-autonomous exhaustion of adult DG NSCs, along with impaired migration in cultured NSCs and reduced proximity of NSCs to local blood vessels in mouse DG. Our findings support an exclusively intracellular mechanism for VEGF signaling in adult DG NSCs, thereby providing resolution to previously conflicting studies and suggesting that cellular source can dictate the functional impact of soluble ligands in DG NSCs.

**Supplementary Information:**

The online version contains supplementary material available at 10.1007/s12035-025-04861-1.

## Introduction

The dentate gyrus (DG) of the hippocampus is one of a few isolated regions in the adult mammalian brain where neural stem and progenitor cells (NSPCs) reside and proliferate throughout life [1]. The newborn neurons generated by these cells support both hippocampal memory and affect regulation [2, 3]. Balancing the preservation of a quiescent radial glia-like neural stem cell (RGL-NSC) pool and active proliferation to generate intermediate progenitors (IPCs) is essential for the lifelong production of new neurons to support hippocampal function [4, 5].

Vascular endothelial growth factor (VEGF) signaling has long been implicated in the preservation of adult hippocampal neurogenesis. Early work by Fabel et al. [6] and Cao et al. [7] suggested that VEGF served as a primary molecular driver of cell proliferation in response to stimuli such as exercise and environmental enrichment. Subsequent studies from the late Ron Duman’s lab more firmly implicated VEGF receptor 2 (VEGFR2) signaling in promoting NSPC proliferation both in healthy rodents [8] and in rodent models of seizure [9] and depression [10]. Our own work has more specifically implicated NSPCs as an essential source of VEGF that promotes their own maintenance via self-stimulated VEGFR2 signaling. Specifically, we previously showed that the knockdown of VEGF in adult NSPCs led to the exhaustion of the DG RGL-NSC pool [11], an effect that could be mimicked in isolated, cultured NSCs and appeared to rely on VEGFR2. We also showed more recently that NSPC-derived VEGF is essential for maintaining NSPC proximity to local blood vessels in the adult DG, most likely via VEGFR2-dependent stimulation of cell motility and attachment [12].

In contrast to the above research, there are several conflicting lines of evidence about the role of VEGF in regulating NSPCs. Relevant to the idea that NSPCs rely on self-generated VEGF, our own work has shown that while NSPCs express VEGF, they do so to a lesser extent than local astrocytes [12]. Given that astrocytes are slightly more abundant than NSPCs in the adult DG [13], it is then puzzling why NSPCs would rely so heavily on self-synthesized VEGF when astrocyte-derived VEGF would, in theory, be abundant. In addition, several studies have suggested that VEGF may not play any role in NSPC regulation. Specifically, neutralization of VEGF in the DG had no effect on basal cell proliferation or neurogenesis [14] and the promotion of adult neurogenesis by VEGF overexpression relied on indirect mechanisms such as angiogenesis or microglial activation [15, 16]. Taken as a whole, the existing literature paints an uncertain picture about how or even whether VEGF regulates adult RGL-NSCs and adult neurogenesis.

Implicit in previous work, including our own, is the assumption that VEGF initiates signaling via activation of cell surface receptors after its secretion into the extracellular milieu. While VEGF is indeed a secreted, soluble protein, it also has been reported to maintain stemness in several non-neural stem cell populations in a cell-autonomous fashion, signaling in an intracellular autocrine (intracrine) loop in cells that express both VEGF and VEGFR2 [17–19]. Notably, an intracrine VEGF signaling loop within DG RGL-NSCs could offer a parsimonious explanation for the seemingly disparate findings in the field. Whether such a loop is operative in DG RGL-NSCs remains unclear.

In this study, we investigated the cellular signaling mechanism by which VEGF maintains RGL-NSC quiescence and motility both in cultured adult DG NSCs and within the adult rodent DG. We found that cultured adult DG NSCs exhibited an active intracrine VEGF-VEGFR2 signaling loop. We also found that NSCs actively cleaved VEGFR2 from their cell surface and were insensitive to extracellular VEGF levels. Intracrine VEGF signaling was essential for maintaining stemness and cell motility in cultured NSCs. In intact mice, we further showed that cell-autonomous VEGF signaling was required for the maintenance of quiescence of DG RGL-NSCs and for the preservation of their proximity to local blood vessels. These findings strongly suggest that RGL-NSCs respond to VEGF exclusively via intracrine VEGFR2 activation and help to reconcile previous contradictory findings based on their methodology targeting either intracellular VEGFR2 signaling within RGL-NSCs or extracellular VEGF availability.

## Results

### VEGF and VEGFR2 Are Co-expressed in Single RGL-NSCs

**Fig. 1:**
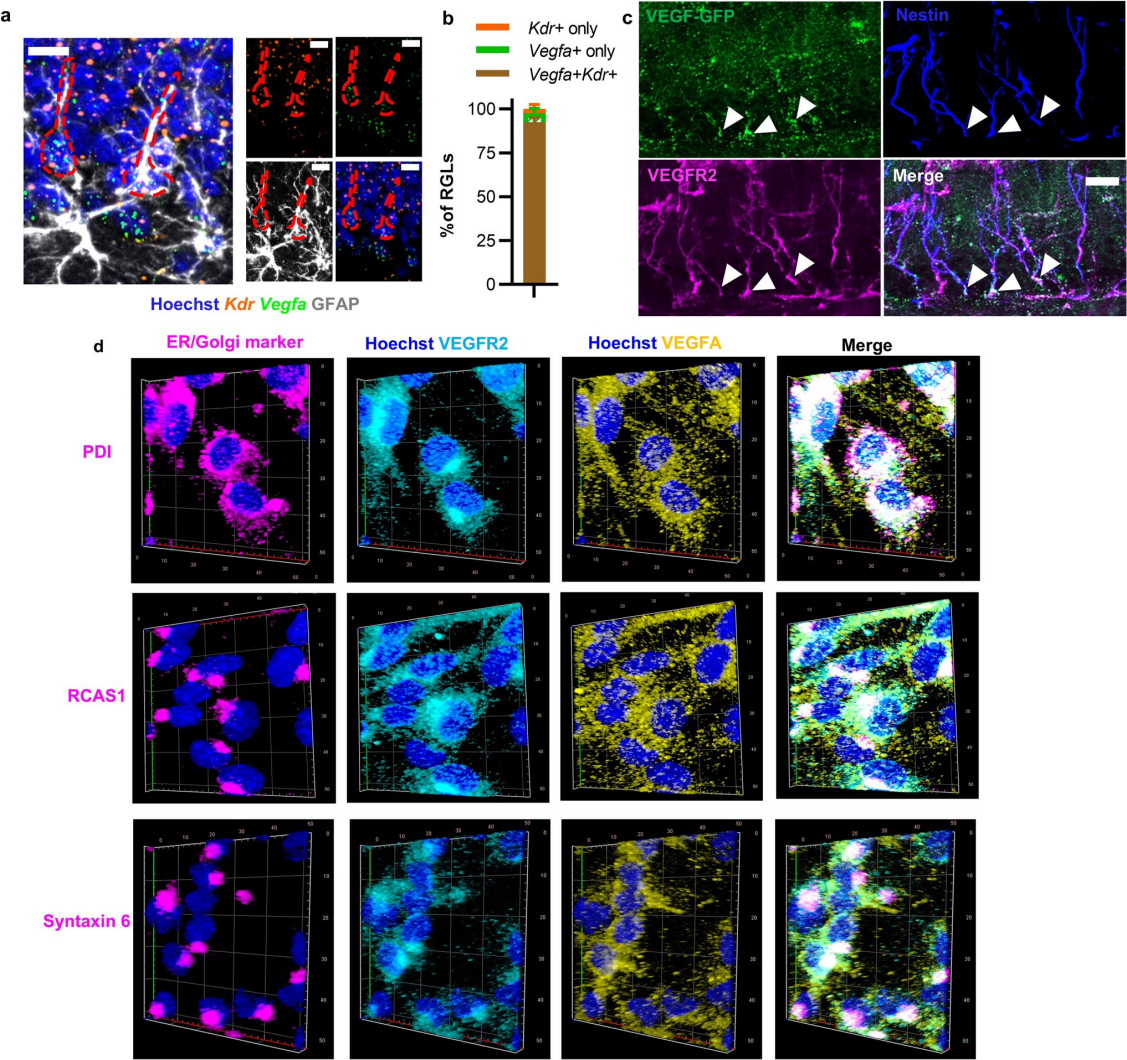
VEGFR2 and VEGF are expressed in the same NSCs. **a** *Kdr* and *Vegfa* in situ hybridization co-labeled with GFAP antibody in adult mouse DG. Scale bar represents 10 µm. **b** Percent of putative RGL-NSCs that are Kdr^+^, *Vegfa*^+^, or *Kdr*^+^/*Vegfa*^+^ by in situ hybridization coupled with immunolabeling for GFAP. *N* = 4 mice; mean ± SEM. **c** Representative immunofluorescent image of VEGFR2 immunolabeling with Nestin in a VEGF-GFP mouse. Arrowheads show VEGFR2^+^/VEGFGFP^+^/Nestin^+^ co-expression. Scale bar represents 20 µm. **d** Representative 3D renderings of z-stack images of VEGFA and VEGFR2 immunoreactivity in the ER (PDI) or Golgi (RCAS1, Syntaxin) in permeabilized cultured NSCs. Hoechst labels nuclei. Major lines in image boxes are 10 µm apart

A requirement for intracrine signaling is that cells express both ligand and receptor simultaneously. RGL-NSCs in mouse DG and in culture express VEGFR2, the primary signaling receptor for VEGF in mammals [11, 12]. To assess co-expression of VEGF and VEGFR2 at the transcriptional level, we used RNAscope in situ hybridization with probes against *Vegfa* and *Kdr* in fixed adult mouse brain sections, coupled with immunolabeling for GFAP. GFAP is a structural protein expressed in adult RGL-NSCs and astrocytes. RGL-NSCs can be distinguished from astrocytes using their unique morphologies: a single GFAP^+^ apical process in RGL-NSCs versus numerous stellate GFAP^+^ processes in astrocytes. Almost all (92.5%) putative RGL-NSCs co-expressed both *Vegfa* and *Kdr* within the same cell (Fig. 1a,b, Online Resource 1A). To further assure co-expression using different markers, we next used Nestin immunolabeling to identify RGL-NSCs in a VEGF-GFP transcriptional reporter mouse in which GFP is downstream of 2.85 kb of the VEGF promoter and 5′ UTR. We have previously demonstrated that the VEGF-GFP signal is abundant in RGL-NSCs [11, 20]. Nestin is a structural protein that is readily identifiable in the primary apical process of RGL-NSCs. We co-labeled VEGF-GFP brain sections for Nestin and VEGFR2. The VEGFR2 antibody selected has been previously knockout validated [21] and we have previously shown that it labels vasculature (as expected) as well as Nestin^+^ NSCs [12]. Here, we added to these previous findings by showing prominent immunoreactivity for both VEGFR2 and VEGF-GFP in single Nestin^+^ RGL-NSCs (Fig. 1c).

To begin to explore the intracellular locations of VEGF and VEGFR2 protein, we used cultured NSCs derived from the adult mouse DG. Cultured DG NSCs are composed predominantly of quiescent and cycling NSCs, as shown using immunolabeling and single-cell transcriptional profiling in our previous work [22]. These NSCs are maintained indefinitely in a defined medium that does not include exogenous VEGF, making NSCs themselves the only source of VEGF in these conditions. We immunolabeled cultured DG NSCs for VEGFA, VEGFR2, and markers of the ER or Golgi: PDI (an enzyme specific to the ER compartment), RCAS1 (a Golgi transmembrane protein) [23–25], or syntaxin-6 (a protein essential for Golgi endosomal trafficking) [26, 27]. Because both VEGF and VEGFR2 are trafficked for extracellular presentation, these proteins would be expected to be found in the ER and Golgi, as well as throughout the cytoplasm. As expected, immunolabels for both VEGF and VEGFR2 were found at some level in the ER and Golgi, as well as in non-ER/Golgi cytoplasm in all observed cells (Fig. 1d). As expected, VEGF and VEGFR2 were generally sparse in the nuclear compartment. These findings provide qualitative evidence of both VEGF and VEGFR2 in the expected intracellular compartments where they could possibly interact.

### Cultured DG NSCs Show Phospho-signaling Dependent on Intracellular but not Extracellular VEGF

**Fig. 2:**
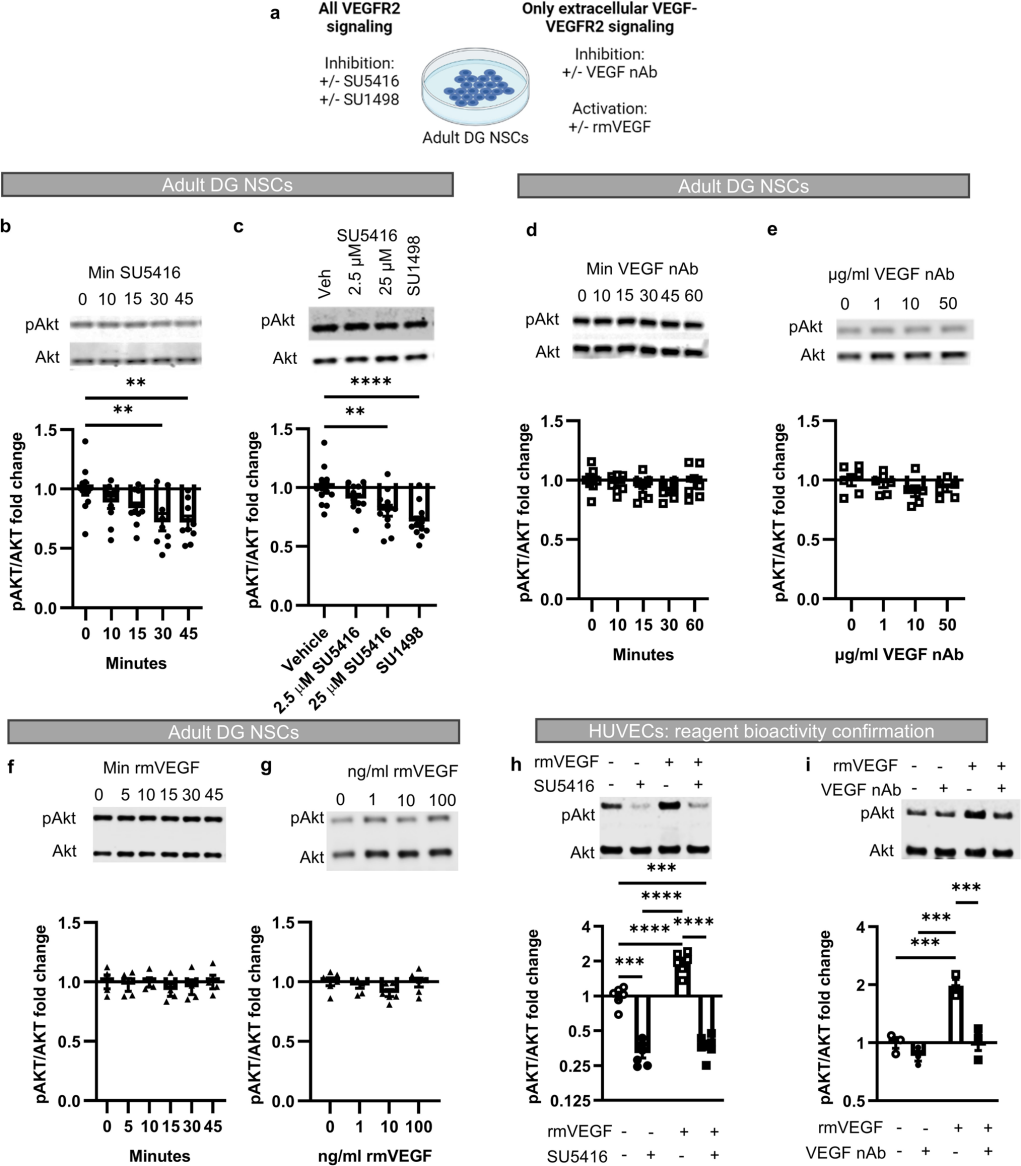
Phospho-signaling downstream of VEGFR2 is insensitive to extracellular VEGF. **a** Experimental paradigm diagram. **b** Representative western blot of Akt phosphorylation in cultured NSCs after SU5416 over time (top). Fold change in Akt phosphorylation after SU5416 over time (bottom). *N* = 1–3/grp/exp, 3 exps; mean ± SEM. **c** Representative western blot of Akt phosphorylation in cultured DG NSCs after treatment with increasing doses of SU5416 or SU1498 (top). Fold change in Akt phosphorylation after SU5416 or SU1498 treatment in cultured NSCs (bottom). *N* = 2–3/grp/exp, 3 exps; mean ± SEM. **d** Representative western blots of Akt phosphorylation in cultured NSCs after VEGF nAb over time (top). Fold change in Akt phosphorylation after VEGF nAb over time (bottom). *N* = 1–3/grp/exp, 3 exps; mean ± SEM. **e** Representative western blot of Akt phosphorylation in cultured DG NSCs after treatment with increasing dosages of VEGF nAb (top). Fold change in Akt phosphorylation after VEGF nAb treatment in cultured NSCs (bottom). *N* = 2–3/grp/exp, 3 exps; mean ± SEM. **f** Representative western blot of Akt phosphorylation in cultured NSCs after recombinant mouse (rm)VEGF treatment over time. Fold change in Akt phosphorylation after rmVEGF over time. *N* = 3/grp/exp, 2 exps; mean ± SEM. **g** Representative western blot of Akt phosphorylation in cultured NSCs after treatment with recombinant mouse (rm)VEGF at increasing doses (top). Fold change in Akt phosphorylation after VEGF treatment in cultured NSCs (bottom). *N* = 3/grp/exp, 2 exps; mean ± SEM. **h** Representative western blot of Akt phosphorylation in cultured HUVECs after treatment with recombinant mouse VEGF and SU5416 (top). Fold change in Akt phosphorylation after VEGF and SU5416 treatment in cultured HUVECs (bottom). *N* = 2–3/grp/exp, 1–2 exps; mean ± SEM. **i** Representative western blot of Akt phosphorylation in cultured HUVECs after treatment with recombinant VEGF and VEGF nAb (top). Fold change in Akt phosphorylation after VEGF and VEGF nAb treatment in cultured HUVECs (bottom). *N* = 3/grp/exp, 1 exp; mean ± SEM. **p < 0.01; ***p < 0.001; ****p < 0.0001

To determine whether DG NSCs use an intracrine VEGF signaling loop, we adopted a common approach for testing intracrine versus autocrine/paracrine signaling: comparing the effects of intracellular receptor inhibition versus extracellular ligand neutralization in cultured cells [19, 28]. We used phosphorylation of a known signaling pathway downstream of VEGFR2, PI3K/AKT, as an assay for VEGFR2 signaling because VEGFR2 phosphorylation itself requires affinity purification with a high degree of receptor concentration to be detectable. Akt signaling was chosen over the other major VEGFR2 downstream signaling partner, MAPK/ERK, because pErk signaling has been previously shown to depend entirely on exogenous FGF2 signaling, while pAkt signaling persisted in the absence of FGF2, suggesting other signals may contribute to its maintenance [29]. Treatment with the cell permeable VEGFR2 inhibitor SU5416 or a separate cell-permeable inhibitor, SU1498, each significantly inhibited phosphorylation of Akt (Fig. 2a–c, Online Resource 2). Consistent with an intracrine signaling mechanism, the VEGF neutralizing antibody (nAb) that specifically binds extracellular VEGF did not alter pAkt (Fig. 2d,e). Similarly, adding exogenous recombinant mouse VEGF to the extracellular media did not alter Akt phosphorylation (Fig. 2f,g). To confirm the biological activity of our reagents, we performed similar experiments in human umbilical vein endothelial cells (HUVECs), which are sensitive to both extracellular and intracrine stimulation of VEGFR2. Both SU5416 and VEGF nAb blocked recombinant mouse VEGF-induced increases in pAkt in HUVECs, confirming that these reagents are bioactive and capable of preventing VEGF signaling through the PI3K/AKT pathway (Fig. 2h,i). Together, these data support an intracrine signaling mechanism where NSC-derived VEGF signals exclusively via cell-internal VEGFR2.

### DG RGL-NSCs Lack Cell Surface VEGFR2 in Intact Mouse DG and in Culture

**Fig. 3:**
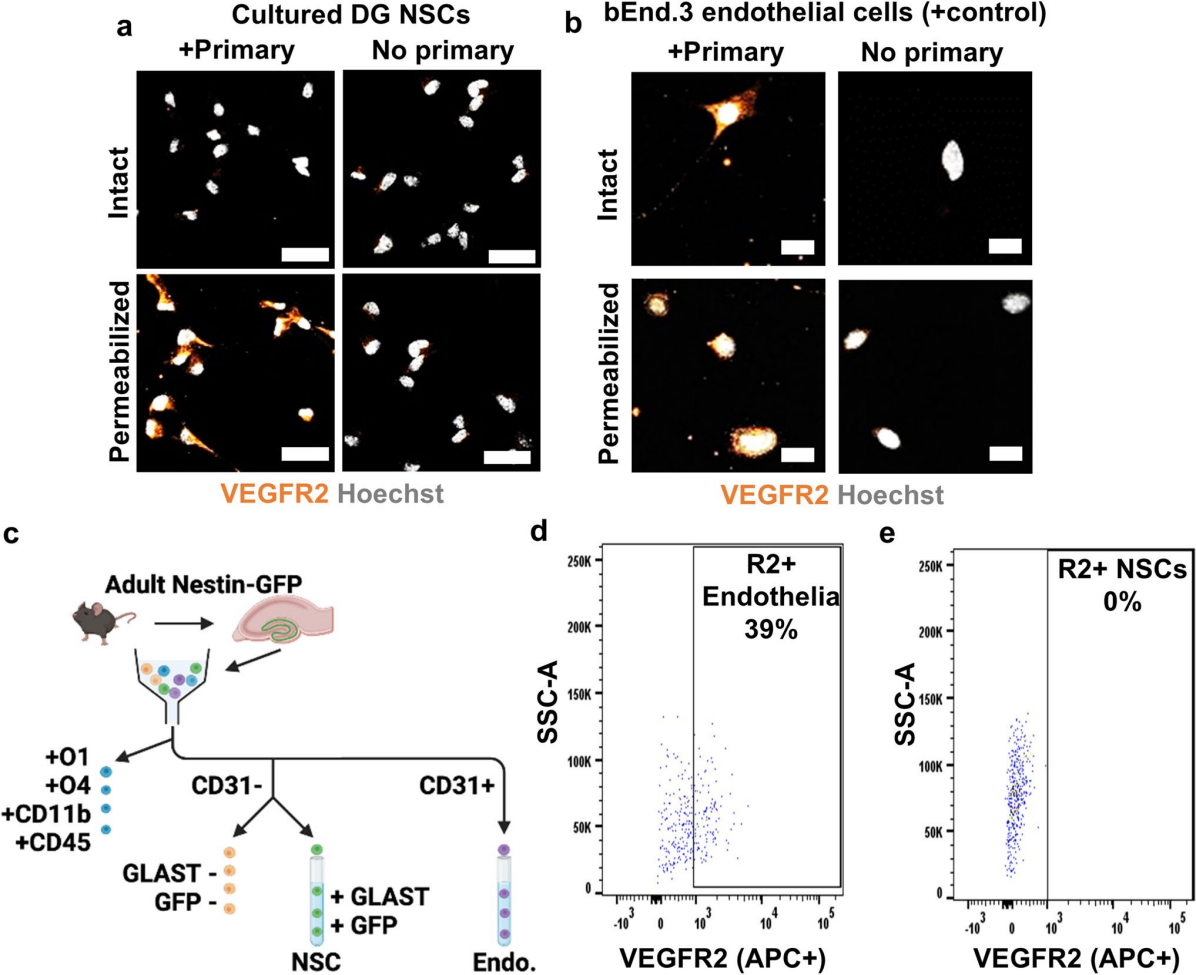
NSCs lack immunoreactive VEGFR2 on the cell surface in culture and in acutely isolated cells. **a** VEGFR2 immunoreactivity of permeabilized and non-permeabilized (intact) cultured DG NSCs in conditions with VEGFR2 primary antibody present or not (no primary). Scale bar represents 20 µm. **b** Representative VEGFR2 immunoreactivity in permeabilized and intact cultured bEnd.3 cells in conditions with VEGFR2 primary antibody present or not (no primary). **c** Diagram of flow cytometry design. **d**, **e** Representative flow cytometry plot of cell surface VEGFR2^+^ (APC^+^) labeling in CD31^+^ endothelial cells and NSCs

One reason intracrine signaling can dominate in a cell that expresses both ligand and receptor is the absence of the receptor on the cell surface. We therefore next investigated whether VEGFR2 may not be at the cell surface of cultured NSCs, as would be expected. Using immunocytochemical staining of non-permeabilized (intact) and permeabilized cultured DG NSCs, we confirmed that only permeabilized NSCs showed immunoreactivity with an antibody targeting the N-terminus of VEGFR2, suggesting that the N-terminal VEGF binding sites of VEGFR2 are present only within the intracellular compartment of cultured NSCs (Fig. 3a). In contrast to NSCs, cultured mouse brain endothelial cells (bEnd.3), which are well established to respond to extracellular VEGF, showed N-terminal VEGFR2-immunoreactivity both on the cell surface and within the intracellular compartment (Fig. 3b). To confirm the specificity of this N-terminal targeting antibody against VEGFR2, we designed a vector for transient CRISPRi suppression of Kdr expression (αVEGFR2). Transient transfection with this vector in bEnd.3 cells resulted in a significant suppression of Kdr expression compared to a control vector carrying a non-target single guide RNA (NT) as quantified by real-time qPCR (Online Resource 1B). Transient transfection with the αVEGFR2 vector, which also carried a GFP reporter, reduced both intracellular and extracellular VEGFR2 immunoreactivity in bEnd.3 cells and intracellular VEGFR2 immunoreactivity in NSCs compared to NT-transfected cells (Online Resource 1C-E), confirming the specificity of the VEGFR2 antibody.

To examine VEGFR2 cell surface expression in acutely isolated RGL-NSCs, we used VEGFR2 immunolabeling and flow cytometry in acutely dissected DG cell suspensions from adult NestinGFP mice (Fig. 3c). Based on our published protocol [30], RGL-NSCs can be identified in these mice as O1^−^O4^−^CD11b^−^CD45^−^CD31^−^GLAST^hi^GFP^+^ cells (Online Resource 1F). We also examined CD31^+^ endothelia as a positive control, where VEGFR2 cell surface labeling should be evident. We found that VEGFR2 was readily detectable on the cell surface of endothelia (31.80% + / − 4.70% R2^+^) but was not detectable on the surface of NSCs (0.14% + / − 0.06% R2^+^) (Fig. 3d,e). When we attempted similar immunolabeling as above in permeabilized cells, antibody labeling was not present in either NSCs or endothelial cells, suggesting that the VEFGR2 antibody was not effective in these conditions. However, VEGFR2 labeling was evident in our previous data from fixed, permeabilized tissue sections (Fig. 1c). Taken together, these findings suggest that RGL-NSCs express VEGF and VEGFR2 but do not localize VEGFR2 on the cell surface, either in culture or in cells acutely isolated from mouse DG.

### NSCs Express Sheddases Which Cleave VEGFR2 from the Cell Surface

**Fig. 4:**
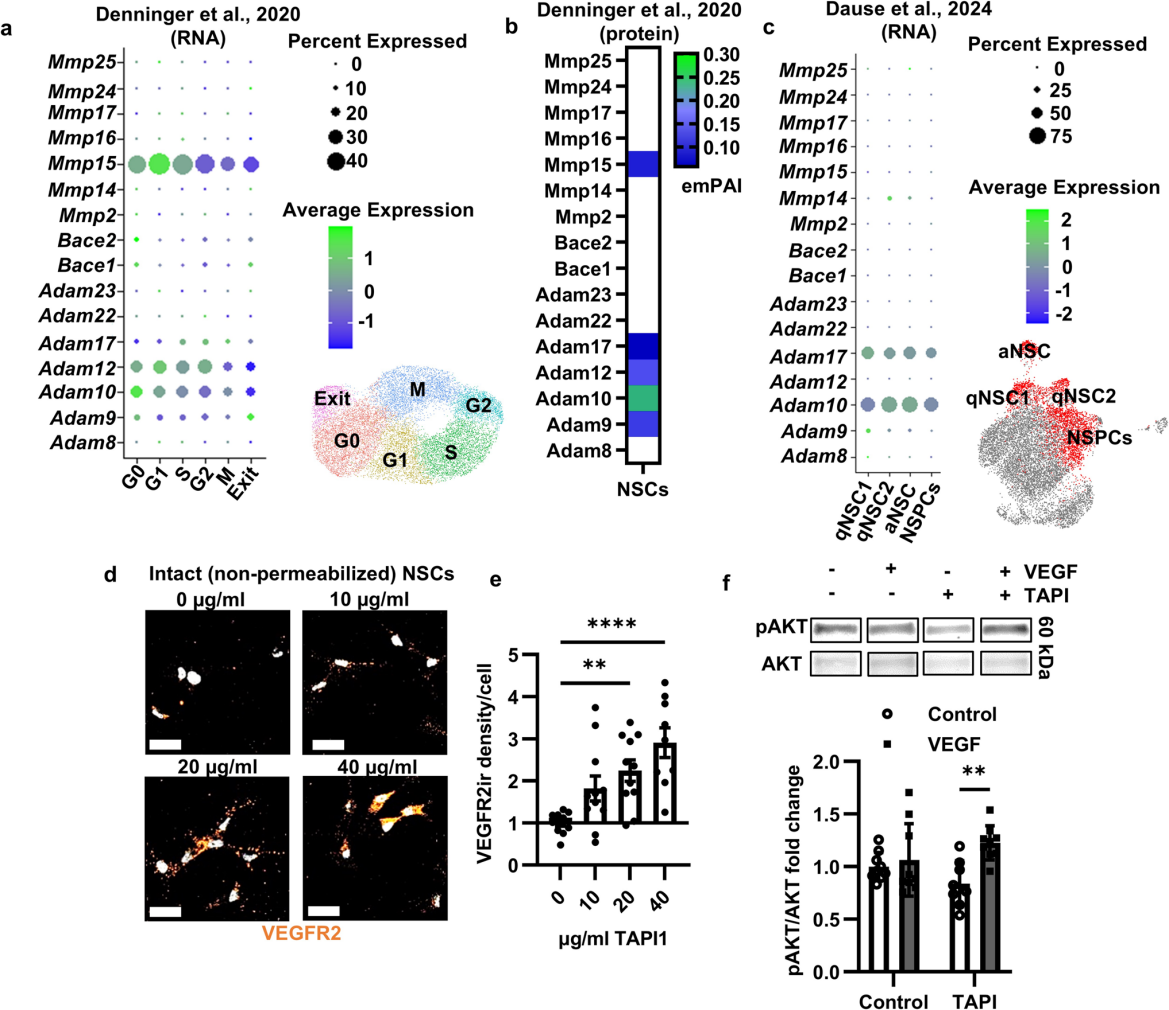
Sheddases cleave VEGFR2 from the cell surface of NSCs in culture. **a**, **b** Sheddase RNA counts from scRNAseq (**a**) and protein expression (emPAI) from LC–MS/MS (**b**) of cultured DG NSCs in [22]. **c** Sheddase RNA expression from scRNAseq of acutely isolated adult hippocampal EYFP^+^ NSCs of NestinCreER.^T2^;LoxStopLox-EYFP mice in [12]. **d** Representative VEGFR2 immunoreactivity in intact cultured NSCs following TAPI-1 treatment. Scale bars represent 20 µm. **e** Mean cell surface VEGFR2 immunoreactivity per cell following TAPI-1 treatment in cultured NSCs. *N* = 2–3 wells/exp, 4 exps; mean ± SEM. **f** Representative western blots of Akt phosphorylation in cultured NSCs after TAPI-1 and/or recombinant mouse VEGF treatment (top). Fold change in Akt phosphorylation after TAPI and/or recombinant VEGF treatment (bottom). *N* = 3/grp/exp, 3 exps; mean ± SEM and individual wells shown. **p < 0.01. ****p < 0.0001

One common cause of insensitivity to extracellular soluble signals is the cleavage of cell surface receptors by extracellular sheddases after insertion into the membrane. Sheddases are membrane-bound enzymes that cleave transmembrane proteins, consisting of members from the ADAM, BACE, and MMP protein families. Analysis of our own previously published single-cell RNA sequencing and liquid chromatography tandem mass spectrometry data from cultured adult DG NSCs revealed that multiple sheddases are detectable throughout the cell cycle and in quiescent NSCs, particularly *Mmp15*, *Adam9*, *Adam10*, and *Adam12* (Fig. 4a,b) [22]. To examine RGL-NSCs without long-term culturing, we analyzed single-cell RNA sequencing from cells acutely isolated from the DG of adult NestinCreERT2^+/−^;LoxStopLoxEYFP mice after tamoxifen injection to activate EYFP expression in RGL-NSCs and their progeny [12]. In clusters containing RGL-NSCs, we found the expression of multiple sheddases, including *Adam10* and *17* (Fig. 4c). These findings suggest broad sheddase expression in NSCs in mouse DG and in culture.

To determine whether sheddases cleave VEGFR2 in DG NSCs and thereby prevent activation by extracellular VEGF, we treated cultured NSCs with tumor necrosis factor-α protease inhibitor 1 (TAPI-1), a broad-spectrum inhibitor of sheddases. TAPI-1 treatment significantly increased VEGFR2 immunoreactivity on the cell surface of cultured NSCs (Fig. 4d,e). To determine whether the preservation of VEGFR2 on the cell surface was functional, we treated NSCs with TAPI-1 while supplementing exogenous rmVEGF in the media and then quantified the Akt phosphorylation response. While vehicle-treated cells showed no pAkt response to rmVEGF (as we found in Fig. 2f,g), TAPI-1 treatment restored the pAkt response to exogenous rmVEGF (Fig. 4f). Together, these data suggest that sheddase cleavage of VEGFR2 from the NSC cell surface inhibits the NSC response to extracellular VEGF.

### Cultured NSCs Rely on Cell Autonomous VEGF to Support Motility

**Fig. 5:**
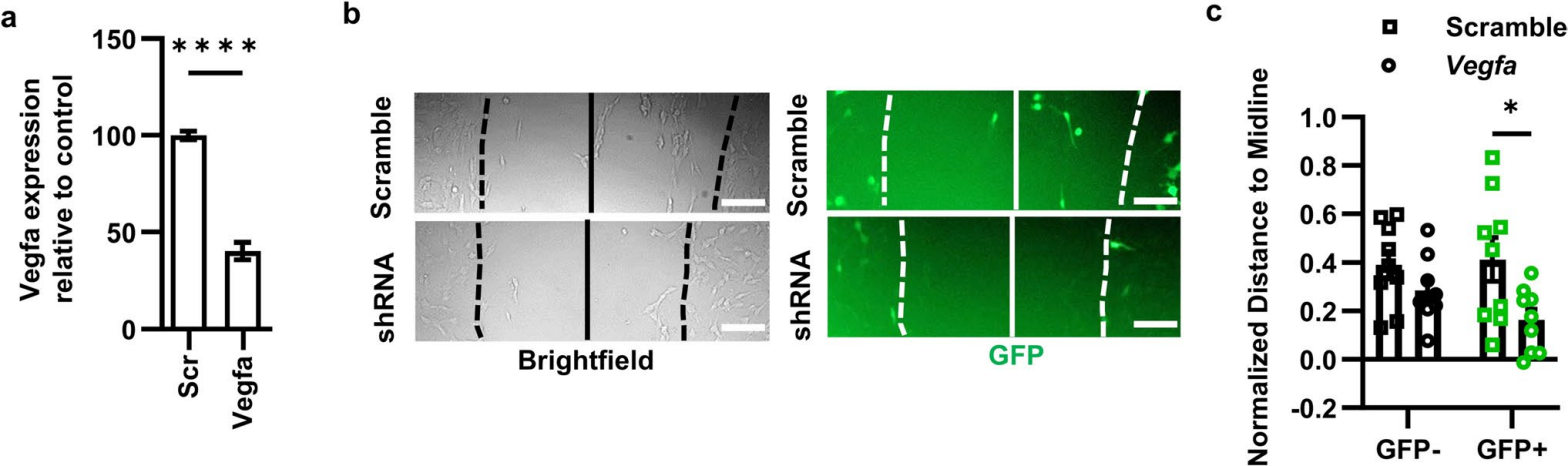
Loss of VEGF expression cell autonomously impairs NSC migration in culture. **a** Relative VEGF protein in conditioned media of WT NSCs after Scramble or *Vegfa* shRNA infection. *N* = 3/grp/exp, 2 exps; mean ± SEM. **b** Representative images of total (brightfield) and GFP^+^ NSC migration into the scratch following treatment with *Vegfa* shRNA or control virus after 8 h. Solid line = scratch midline. Dotted line = scratch border. Scale bars represent 100 µm. **c** Comparison of GFP^+^ and GFP^−^ cell distance to the midline normalized to initial scratch width such that no movement towards the center would be 0 and reaching the midline would be 1. *N* = 3 wells/experiment, 3 experiments. Mean ± SEM and individual wells shown. *p < 0.05, ****p < 0.0001

We previously showed, using VEGFR2 inhibition in a scratch assay, that cultured DG NSCs rely on VEGFR2 signaling to maintain motility in culture [12]. To determine whether this effect relied on cell-autonomous VEGF signaling, we infected cultured NSCs with lentiviral vectors expressing GFP with either a shRNA against *Vegfa* or a scrambled shRNA control. In a separate assay, we first confirmed that *Vegfa* shRNA led to a 60 ± 11% suppression of VEGF compared to scrambled shRNA (Fig. 5a). Next, we performed a scratch assay, then imaged GFP^+^ and GFP^−^ NSC migration into the scratch. Migration of GFP^+^ NSCs was impaired in *Vegfa* shRNA-treated NSCs compared to scrambled-treated NSCs (Fig. 5b,c). GFP^−^ cells, however, showed no effect of shRNA treatment (Fig. 5b,c). These findings suggest that the loss of VEGF impacts NSC migration in a cell-autonomous manner.

### Cultured NSCs Rely on Cell Autonomous VEGF to Prevent Exhaustion

**Fig. 6:**
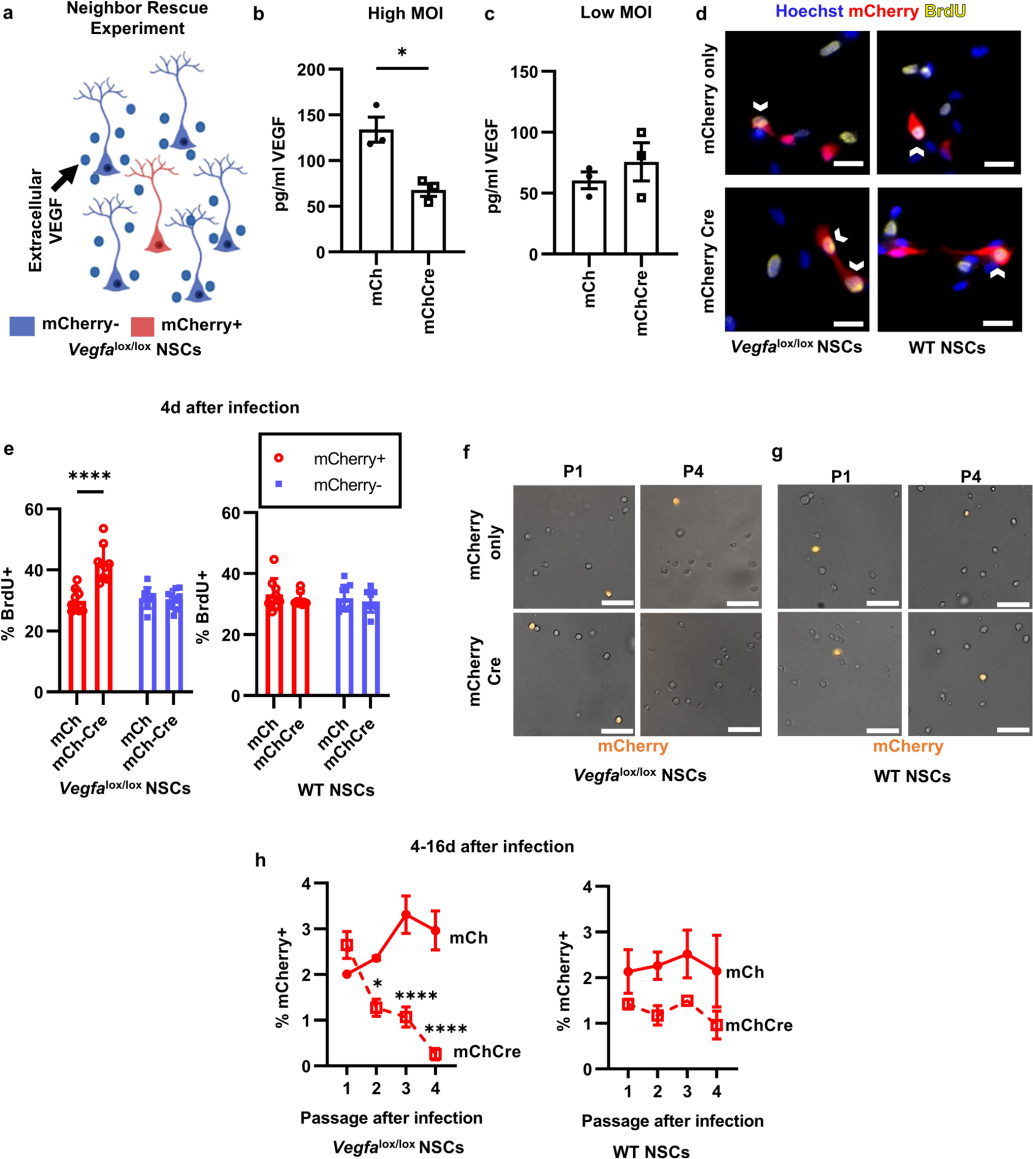
Cell autonomous VEGF signaling maintains NSCs in culture. **a** Diagram of the neighbor rescue experiment. **b**, **c** VEGF concentration (pg/ml) in NSC conditioned media after treatment with high MOI (**b**) or low MOI (**c**) mCherry or mCherryCre lentivirus. *N* = 3 wells/grp; mean ± SEM plus individual wells shown. **d** Representative immunofluorescent images of BrdU^+^ cultured *Vegfa*^lox/lox^ and WT NSCs (Hoechst^+^) after lentiviral infection (mCherry^+^). Chevrons indicate BrdU^+^ mCherry^+^ Hoechst^+^ NSCs. **e** Percent of mCherry^+^ or mCherry^−^ *Vegfa*^lox/lox^ and WT NSCs that were BrdU^+^ after low MOI mCherryCre and mCherryonly lentiviral infection. *N* = 3/grp/exp, 3 exps. Mean ± SEM shown. **f** Representative images of cultured *Vegfa*^lox/lox^ NSCs (brightfield) after lentiviral infection (mCherry^+^) after 1 and 4 passages. Images taken immediately after passage, when counting was performed. **g** Representative images of cultured WT NSCs (brightfield) after lentiviral infection (mCherry^+^) after 1 and 4 passages. Images taken immediately after passage, when counting was performed. **h** Percent of mCherry^+^ *Vegfa*^lox/lox^ and WT NSCs after infection with low MOI mCherryCre or mCherryonly lentiviral vectors. *N* = 3/grp/exp, 2 exps; mean ± SEM. Scale bars represent **d** 10 µm and **f**, **g** 50 µm. *p < 0.05; **p < 0.01; ****p < 0.0001

We previously showed that adult DG NSCs in culture require VEGF/VEGFR2 signaling to prevent over-activation and subsequent exhaustion [11]. To determine whether this effect relies on cell-autonomous/intracrine VEGF, we used a neighbor rescue design in cultured NSCs derived from adult DG of *Vegfa*^lox/lox^ mice (Fig. 6a). We infected *Vegfa*^lox/lox^ NSCs with a mCherryCre lentiviral vector to knockdown VEGF (or mCherryonly as control). High multiplicity of infection (MOI) mCherryCre infection led to a significant suppression of VEGF in NSC conditioned media compared to mCherryonly control (Fig. 6b), verifying the effectiveness of the lentiviral knockdown. To study individual VEGF knockdown versus intact cells, we next infected cells with a low MOI of lentiviral particles, resulting in less than 5% of NSCs showing viral expression (mCherry). At the low MOI, extracellular VEGF was not altered (Fig. 6c). The low MOI condition, therefore, provided a model where the infrequent mCherry^+^ cells have lost cell-internal VEGF yet still are exposed to the presence of abundant extracellular VEGF from neighboring cells.

The exhaustion of cultured NSCs is characterized by an early phase of over-proliferation (days) followed by diminished self-renewal over the long term (weeks). To investigate the early over-proliferation phase, we treated *Vegfa*^lox/lox^ NSCs with low MOI mCherryCre for 4 days, then pulsed with BrdU to label proliferating NSCs. Low MOI mCherryCre treatment led to an acute increase in BrdU^+^ proliferative cells selectively among mCherry^+^ cells, while mCherryonly expression had no effect on proliferation (Fig. 6d,e). There was no effect of mCherryCre versus mCherryonly expression in wildtype (WT) NSCs (Fig. 6d,e). When *Vegfa*^lox/lox^ NSCs were grown over multiple passages to investigate the long-term loss of self-renewal, a complete and selective loss of mCherryCre^+^ NSCs was observed, while mCherryonly infected NSCs were preserved (Fig. 6f–h). There was no effect of mCherryCre versus mCherryonly expression in wildtype (WT) NSCs (Fig. 6f–h). These findings suggest that cell autonomous VEGF signaling is necessary to prevent NSC activation and subsequent exhaustion.

### RGL-NSCs in Intact DG Rely on Cell-Autonomous VEGF to Maintain Quiescence and Proximity to Local Blood Vessels

**Fig. 7:**
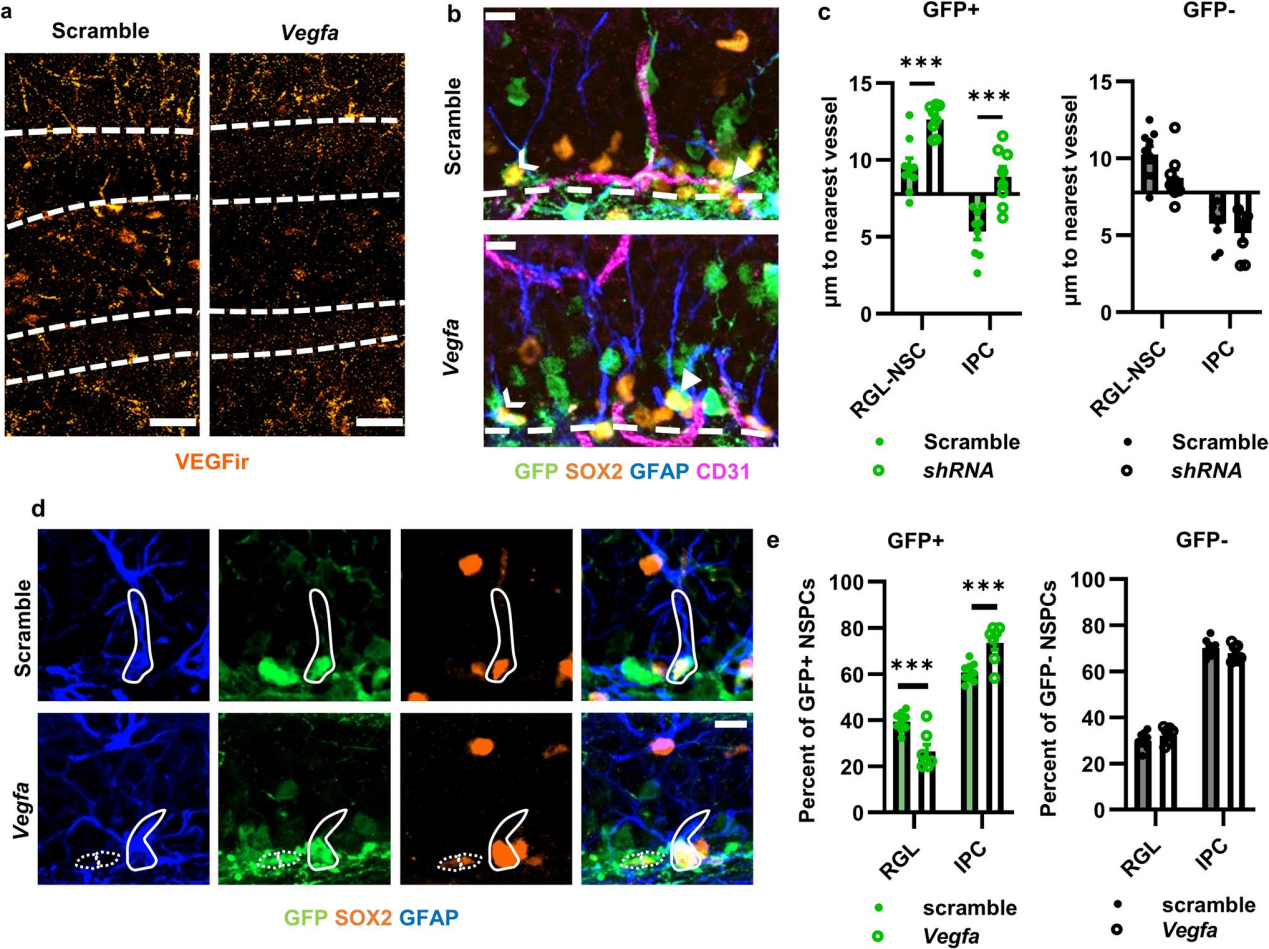
Suppression of VEGF expression cell autonomously reduces vessel association and RGL-NSC maintenance in the adult DG. **a** Representative images of VEGF immunolabeling in the DG of scramble and shRNA treated mice. **b** Representative immunofluorescent images of shRNA expressing (GFP^+^) or non-expressing (GFP^−^) GFAP^+^SOX2^+^ RGL-NSCs and GFAP^−^SOX2^+^ IPCs and their association with the CD31^+^ vasculature 21 days after viral infusion. Chevrons indicate GFP^+^ RGL-NSCs, and arrowheads indicate GFP^+^ IPCs. Scale bars represent 10 µm. Images reproduced from [12]. **c** Distance from the nearest CD31^+^ vessel for GFP^+^ or GFP^−^ GFAP^+^SOX2^+^ RGL-NSCs or GFAP^−^SOX2^+^ IPCs 21 days after viral infusion. Bars start at the average distance for a random SGZ cell. GFP^+^ cell data reproduced from [12]. **d** Representative images of GFAP^+^SOX2^+^ RGL-NSCs and GFAP^−^SOX2^+^ IPCs. GFP^+^ RGL-NSCs have a solid outline. GFP^+^ IPCs have a dotted outline. Scale bars represent 10 µm. **e** Percent of GFP^+^ and GFP^−^ NSPCs that were GFAP^+^SOX2^+^ RGL-NSCs versus GFAP^−^SOX2.^+^ IPCs 21 days after viral infusion. *N* = 8–9 mice/grp. Mean ± SEM plus individual mice shown. *p < 0.05; ***p < 0.001

Our findings thus far indicated that VEGF signals cell autonomously to support cultured NSC motility and prevent exhaustion, findings that are consistent with an intracrine VEGF-VEGFR2 signaling loop. We next set out to determine if cell-autonomous VEGF signaling is also critical in NSPCs in intact DG. To be able to reliably differentiate cells that have lost VEGF versus neighboring cells that have not, we used lentiviral vectors expressing *Vegfa* (or scramble) shRNA plus GFP. We perfused mice 21 days after stereotaxic viral infusion in the adult DG. We previously reported that *Vegfa* shRNA reduced VEGF immunoreactivity throughout the DG by 74 ± 12% compared to the scramble control in these mice (Fig. 7a) [12]. In this model, all cells are exposed to these broad changes in extracellular VEGF, but *Vegfa* shRNA-expressing GFP^+^ cells also lose cell-internal VEGF. Neighboring GFP^−^ cells, in contrast, retain that cell-autonomous VEGF expression.

We previously linked impaired NSPC migration/adhesion capacity after VEGF signaling loss to reduced proximity to local blood vessels in adult mouse DG [12]. To assess the role of cell autonomous VEGF in this effect, we looked at the distance of RGL-NSCs and IPCs from the nearest blood vessel. RGL-NSCs were identified as GFAP^+^SOX2^+^ cells with cell bodies in the SGZ and an apical process extending through the granule cell layer in fixed brain sections. IPCs were identified as GFAP^−^SOX2^+^ cell bodies in the SGZ. After identification, the distance to the nearest blood vessel was measured using immunolabeling for CD31, an endothelial cell surface protein, to identify vessel structures. After the distance measurement, cells were then categorized as GFP^+^ or GFP^−^. We previously reported that *Vegfa* shRNA expression led to a significant disruption in GFP^+^ RGL-NSC and IPC distance to the vessel in these mice [12]. Here, we expand on that analysis and show that *Vegfa* shRNA had no effect on the distance of neighboring GFP^−^ RGL-NSCs or IPCs to the nearest blood vessel, despite widespread loss of VEGF throughout the DG (Fig. 7b,c). These results suggest that self-synthesized, cell autonomous VEGF is sufficient for the maintenance of NSPC proximity to vasculature.

In mouse DG, RGL-NSC exhaustion after the loss of VEGF is characterized by early activation and symmetric exhaustive divisions of RGL-NSCs, causing a surge in IPC production at ~ 21 days after VEGF loss [11]. Variability in viral infection rates made counts of total GFP^+^ cell numbers unreliable. We therefore quantified the relative percentages of RGL-NSCs and IPCs that were GFP^+^ and GFP^−^ in shRNA infused mice. We found that in *Vegfa *shRNA treated mice, GFP^+^ NSPCs shifted away from the RGL-NSC phenotype in favor of the GFP^+^ IPC phenotype compared to scramble-treated mice. GFP^−^ NSPCs, in contrast, showed similar proportions of phenotypes between *Vegfa* and scramble-treated mice (Fig. 7d,e). These findings suggest that self-synthesized, cell-autonomous VEGF is sufficient for the prevention of RGL-NSC exhaustive differentiation.

## Discussion

Here, we provide evidence for an intracrine VEGF-VEGFR2 signaling loop that is important for maintaining RGL-NSC quiescence and supporting NSPC proximity to local blood vessels. This finding provides an explanation for why RGL-NSCs might depend on their own VEGF despite abundant alternate VEGF sources (e.g., astrocytes). Our finding that RGL-NSCs appear to be insensitive to extracellular VEGF also has important implications when considering strategies to support adult neurogenesis. Specifically, our findings suggest that supplementation with exogenous VEGF may not be effective in providing signals to RGL-NSCs, as we found that shedding of extracellular receptors limited responsivity to extracellular VEGF.

VEGF has previously been shown to support survival and/or quiescence through an intracrine loop in several other cell populations, including hematopoietic stem cells, cancer stem cells, and endothelia [17–19]. In addition to intracrine signaling loops, however, these cells are typically also sensitive to extracellular VEGF [28]. In contrast, we found that DG NSCs are insensitive to extracellular VEGF levels, likely because the extracellular VEGF binding domain of VEGFR2 is cleaved by sheddases on the cell surface. This insensitivity to extracellular VEGF provides substantial resolution for the previously conflicting literature on VEGF and adult neurogenesis. Specifically, studies suggesting no effect of VEGF loss on adult neurogenesis often relied on methods that neutralized extracellular VEGF [14, 16] while studies suggesting that loss of VEGF signaling suppressed adult neurogenesis often relied on long-term infusion of cell-permeable VEGFR2 inhibitors [8–10]. All of these results are consistent with a model where cell-internal VEGF is necessary and sufficient to prevent RGL-NSC exhaustion, and external VEGF does not directly impact RGL-NSCs.

The VEGF intracrine signaling mechanism that we describe here appears to be somewhat unique to NSCs of the adult DG. Though embryonic NSCs are well known to produce and secrete VEGF ligand [31], they do not rely on intracrine or autocrine VEGFR signaling for self-regulation [32]. Rather, embryonic NSC VEGF seems to be critical primarily for stimulating the proliferation and migration of developing vasculature [31]. It also seems unlikely that NSCs in the adult SVZ rely on VEGF intracrine signaling, as we have shown that adult SVZ NSCs express much lower levels of VEGF and VEGFR2 than DG NSCs [12]. The mechanisms by which NSCs in the DG develop reliance on VEGF intracrine signaling to maintain quiescence remain to be investigated, as does the possibility that this shift supports their deepening quiescence over postnatal development. The question of why adult DG NSCs would develop this somewhat unique dependence also remains. One strong possibility, supported by our data and mentioned above, is that they share a reliance on VEGF with other stem cell types (and some cancer stem cells), but their high expression of sheddases eliminates extracellular VEGF signaling, leaving only intracrine signaling to perform the functions of VEGF. Another possibility is that in vivo VEGF levels in the adult DG are too low to provide strong stimuli to NSCs (even if sheddases did not interfere), and a cell-internal VEGF evolved to compensate.

In addition to supporting RGL-NSC quiescence, we also found that cell-autonomous VEGF supported RGL-NSC motility in culture and proximity to local vasculature in DG. The vascular niche of the adult DG is unique, with an exceptionally dense network of planar capillaries being found in the SGZ where NSPC bodies reside [33]. Within this niche, the NSPCs are found especially close to blood vessels [34, 35]. This arrangement is hypothesized to support adult neurogenesis by providing NSPCs with access to circulatory factors and endothelial-derived signals that promote RGL-NSC self-renewal and production of new neurons [33, 36]. This loss of vascular contact presents a confounding variable for determining how VEGF signaling supports RGL-NSC maintenance. In the present study, we cannot determine whether the shift from RGL-NSC phenotype to IPC phenotype after cell-autonomous loss of VEGF is due to loss of a VEGFR2 signaling program that directly supports stemness or whether it is secondary to loss of access to vascular signals. In culture, we were able to observe dependence on cell-autonomous VEGF for both migration and stemness preservation. In vivo, it seems possible that both of these mechanisms could contribute to stem cell maintenance. Future work will be necessary to separate these two mechanisms. We also report here a role for NSPC-derived VEGF in IPC proximity to blood vessels. It is unclear whether this dependence relies on intracrine VEGF in IPCs, paracrine VEGF, or is simply an artifact of IPCs being born from NSCs that were farther from blood vessels. IPCs are also known to migrate towards vessels from their parent NSC [35] and the signals needed to direct that migration remain unclear. Future work is needed to resolve these questions. Imaging of thick, cleared tissue in 3D could also advance our understanding of the NSPC-vascular physical relationship.

There are several limitations to the present study. First, our demonstration of VEGF co-expression with VEGFR2 in single RGL-NSCs here relies mostly on the assessment of transcription of VEGF. Secreted proteins have short intracellular half-lives and are most often identified in immunolabeling in the extracellular space or bound to receptors. It is therefore difficult to determine the cellular source with a VEGF immunolabel. Similarly, the subcellular location of VEGF and VEGFR2 signaling requires more exploration, especially to demonstrate downstream signaling generated from intracellular VEGF-VEGFR2 binding and what percent of NSCs show VEGF-sensitive pAKT signaling. Our immunolabeling shows qualitative overlap of VEGF and VEGFR2 protein in the expected intracellular compartments of the ER, Golgi, and cytoplasm, but more work is required to demonstrate that these two proteins interact in these compartments and in what fraction of NSCs such interaction occurs. We must also note that in RNAscope analyses, RGL-NSCs were identified solely using the morphology of GFAP^+^ processes extending from a Hoechst^+^ nucleus. Though this method follows previously published data [37, 38], RGL identity would be more certain if other co-labels, such as SOX2, were employed. Second, though we focus here on VEGFR2, VEGF binds to several receptors and co-receptors in the rodent brain. Our previous work shows that VEGFR2 is the primary receptor expressed by NSCs [11] and previous findings from our lab and others [6, 7, 39, 40] show low or no VEGFR1 expression in adult DG NSCs. VEGFR1 is also generally thought to function as a decoy receptor because it has little kinase activity of its own. It is therefore often hypothesized to bind to and effectively neutralize extracellular VEGF. Such a role has been described for SVZ NSCs, for example, in which NSC VEGFR1 neutralizes soluble VEGF and limits VEGFR2 signaling in neighboring cells [28]. Still, it is possible that NSC-specific VEGF in the DG may also rely partially on binding to VEGFR1. We also have not addressed here any role for other co-receptors (e.g., neuropilins). Third, more work is needed to better define the downstream mediators of VEGF signaling in adult DG NSCs. While our NSC culture data suggest a role for the PI3K/Akt pathway in VEGF-VEGFR2 intracrine signaling, more work is necessary to confirm kinase pathways downstream of VEGFR2 in intact mouse DG. Finally, in mice and in cultured NSCs, we rely on contrasting viral transgene expressing (reporter +) cells with neighboring cells that do not express the transgene (reporter-) to assess the cell autonomous effects of VEGF loss. This approach cannot prove an intracrine signaling method with complete certainty; observed effects could also be due to secreted VEGF binding receptors exclusively on the cell it was released from, thereby still appearing cell autonomous but not using an intracrine mechanism. Though we control the effect of viral infection itself by using reporter-only controls, it is also possible that some complex interaction of viral infection plus extracellular VEGF loss could contribute to effects. Future research will be needed to develop methods that can manipulate intracrine signaling more specifically to improve on this small uncertainty.

Understanding how RGL-NSCs are preserved throughout the lifespan remains a critical field for both foundational understanding of hippocampal biology and development of regenerative medicine approaches to hippocampal dysfunction. Our findings suggest that VEGF regulation of adult NSC maintenance depends critically on the source of the VEGF ligand. These findings have implications for the design of therapeutics using NSCs, suggesting that exogenous growth factor supplementation may not be fruitful for improving endogenous or transplanted NSC survival. Future studies may investigate how different sources of VEGF or manipulations to VEGR2 expression may preserve neurogenic capacity.

## Methods

### Mice

All animal use was in accordance with institutional guidelines approved by The Ohio State University Institutional Animal Care and Use Committee. Male and female mice 7–10 weeks old were used for experiments. Wild-type C57BL/6 J male and female mice were purchased from Jackson Laboratory (strain #000664) and housed at least 1 week in-house before experiments began. Nestin-GFP mice [41] were also purchased from Jackson Laboratory (strain #033927) and bred in-house. VEGF-GFP mice were a gift from Brian Seed, Harvard University, Cambridge, MA, USA (Fukumura et al., 1998) and were also bred in-house. For culture generation, 6-week-old male and female C57BL/6J mice (Jackson Labs, #000664, purchased at 5 weeks of age) and VEGF^lox^ mice (Genentech, Inc., [42], bred in-house) were used. All mice were housed in standard ventilated cages, with ad libitum access to food and water on a 12-h light cycle, with lights on at 0630. Male and female mice were represented in approximately equal numbers throughout. Analysis shows males and females combined because no sex differences were found in any experiments.

### NSC Culturing

NSCs were derived from adult DG of male and female mice following the protocol of [43]. In brief, mice were euthanized with a ketamine/xylazine mixture (87.5 mg/kg, 12.5 mg/kg, i.p.) and their brains were rapidly extracted and immersed in ice-cold neurobasal A media. The DG was dissected in ice-cold neurobasal A under a dissection scope, as per [44]. DGs were diced with a scalpel and digested with papain-dispase-DNase, as described in [43]. A 22% Percoll gradient was used to remove debris; the cell pellet was washed several times with warm neurobasal A and then resuspended in standard NSC growth media: 20 ng/ml EGF, 20 ng/ml FGF2, 1 × glutamate, 1 × B27 without vitamin A in neurobasal A. Cells were then grown on poly-d-lysine/laminin coated plates. No cells were used past passage 20. Five males were pooled to create a male culture line and five females were pooled to create a female culture line. Validation of these lines is available in [22]. Lines were verified free of mycoplasma.

### Endothelial Cell Culture

Human umbilical vein endothelial cells (HUVEC, Invitrogen) were maintained as per manufacturer instructions on uncoated tissue culture plates. bEnd.3 cells (ATCC) were maintained in Dulbecco’s modified Eagle medium (ATCC) with 10% fetal bovine serum (Fisher) at 37 °C, 5% CO2 in tissue culture dishes.

### RNAscope In Situ Hybridization, Immunohistochemistry, and Analysis

We followed the exact same procedures as in our previous work [12], which we quote here for convenience: “WT C57BL/6 J mice were transcardially perfused with ice cold PBS followed by 4% PFA. Brains were harvested and fixed overnight at 4 °C in 4% PFA before serial overnight equilibration in 10%, 20%, and 30% sucrose. Fixed tissue was snap frozen in OCT in a dry ice/100% ethanol bath and stored at −70 °C. 12 µm cryosections, 1 section per slide, were prepared with a cryostat and stored at −70 °C with desiccant until staining. RNA in situ hybridization was performed according to manufacturer recommendations for using fixed frozen tissue samples in the RNAscope Multiplex Fluorescent v2 Assay (Advanced Cell Diagnostics) with the following modifications to enable concurrent immunohistochemical staining. The pretreatment steps were replaced with a 15 min modified citrate buffer (Dako) antigen retrieval step in a steamer at 95 °C. Additionally, the protease III step was excluded to enable subsequent immunohistochemical staining. Probes for mouse Vegfa (Mm-Vegfa-ver2; ACD) and mouse Kdr (Mm-Kdr-C2; ACD) RNA were hybridized to tissue before subsequent immunohistochemical staining for GFAP protein. Immunostaining for GFAP was conducted as described in (Immunohistochemical tissue processing) with the following exceptions. Blocking was performed with 10% normal donkey serum in 0.1 M tris buffered saline (TBS)−1% bovine serum albumin (BSA). Antibody incubations were performed in TBS-1% BSA. All washes were performed with TBST. DAPI provided by the RNAscope Multiplex Fluorescent kit was used for nuclear counterstaining. All images were acquired with the Zeiss Axio Observer Z1 microscope with Apotome for optical sectioning using a 20 × air objective. Full z-stacks were acquired for analysis. RGL-NSCs and astrocytes were identified based on the morphology of GFAP^+^ apical or stellate processes, respectively, extending from a DAPI^+^ nucleus in 1 μm z-stack images from *n* = 4 mice.”

After a putative RGL-NSC was identified as above, it was assessed for the presence or absence of *Vegfa* and *Kdr* puncta in the nucleus.

### Dentate Gyrus Isolation and Flow Cytometry

DG isolation and flow cytometry was used to identify VEGFR2 surface expression. For VEGFR2 surface expression, 8–10-week-old Nestin-GFP adult mice (*n* = 5) were anesthetized and perfused with HBSS without Ca^2^^+^/Mg^2^^+^. Following perfusions, brains were removed and put in ice-cold HBSS until dissection. Brains were then bisected along the midsagittal line, and overlaying diencephalic structures were removed. DGs were dissected under a dissection microscope as per [44] and placed in ice-cold HBSS without Ca^2^^+^/Mg^2^^+^. DGs were then mechanically dissociated with sterile scalpel blades and then repeatedly with mortar and pestle in douncing buffer on ice. Dissociated cells were collected by centrifugation at 500 g for 5 min before resuspending in HBSS without Ca^2+^/Mg^2+^. Cells were then filtered through a 35-μm nylon filter before staining with fluorescent antibodies (Table 1) on ice for 30 min. During the last 10 min of staining, Hoechst dye was added for live/dead discrimination. All cells were washed twice following staining and immediately processed on the FACS Aria III (BD Biosciences). The data were analyzed using FlowJo™ v10.8.1 software (BD Life Sciences) and NSCs or endothelial populations were identified based on fluorescent markers, with the GFP^+^, Glast^+^, CD31^−^, CD45^−^, CD11b^−^, O1^−^, and O4^−^ live cells designated as NSCs, while CD31^+^, CD45^−^, CD11b^−^, O1^−^, and O4^−^ live cells were designated as endothelia. NSCs and endothelia were then analyzed for VEGFR2 expression.

**Table 1:** List of materials with product numbers

Reagent or resource	Source	Identifier
**Antibodies**		
Cell Signaling Technology, 2920 s, Akt (pan) (WB; 1:100)	Cell Signaling Technology	Cat#2920 RRID: AB_1147620
Cell Signaling Technology phospho-Akt (ser473) (WB; 1:1000)	Cell Signaling Technology	Cat#4060 RRID: AB_2315049
IRDye® 680RD Goat anti-Mouse IgG (H + L), 0.1 mg [P/N 925–68070] (WB; 1:10,000)	VWR	Cat#: 102,971–020 RRID:
IRDye® 800CW Goat anti-Rabbit IgG (H + L), 0.1 mg [P/N 925–32211] (WB; 1:10,000)	VWR	Cat# 103,011–494 RRID:
Hoechst 33,342 (IF; 1:2000) (Flow; 1:5000)	Fisher	Cat#H3570 RRID: N/A
Anti-Glial Fibrillary Acidic Protein, Clone: GA5 (IF; 1:1000)	Sigma	Cat#MAB360 RRID: AB_11212597
Anti-mCherry antibody (IF; 1:500)	Abcam	Cat#ab167453 RRID: AB_2571870
Anti-GFP Antibody (IF; 1:1000)	abcam	Cat#ab6673 RRID: AB_305643
BrdU Antibody (IF; 1:500)	Bio-Rad	Cat# MCA6114 RRID: unknown
Goat anti-VEGF164 (Neutralization; 10 µg/ml) (IF; 1:50)	R&D systems	Cat#AF-493 RRID: AB_354506
SOX2 Rat anti-Human, Mouse, Clone: Btjce (IF; 1:1000)	eBioscience	Cat# 50–112–9095 RRID: unknown
Anti-NeuN, clone A60 (IF; 1:500)	Millipore	Cat#MAB377 RRID: AB_2298772
Rat anti-VEGFR2 (IF in culture cell surface/intracellular labeling; 1:40)	R&D systems	Cat#MAB4432 RRID: AB_2298772
Goat anti-VEGFR2 (IF in slices; 1:100)	R&D systems	Cat#AF644 RRID: AB_355500
PDI (C81H6) Rabbit mAb (IF; 1:500)	Cell Signaling Technology	Cat#3501 RRID: AB_2156433
RCAS1 (D2B6N) XP® Rabbit mAb (IF; 1:500)	Cell Signaling Technology	Cat#12,290 RRID: AB_2736985
Syntaxin 6 (C34B2) Rabbit mAb (IF; 1:500)	Cell Signaling Technology	Cat#2869 RRID: AB_2196500
Donkey anti-Goat IgG (H + L) Cross-Adsorbed Secondary Antibody, Alexa Fluor 488 (IF; 1:500)	Fisher	Cat#A-11055 RRID: AB_2534102
Donkey anti-Rabbit IgG (H + L) Highly Cross-Adsorbed Secondary Antibody, Alexa Fluor 555 (IF; 1:500)	Fisher	Cat#A-31572 RRID: AB_162543
Donkey anti-Goat IgG (H + L) Cross-Adsorbed Secondary Antibody, Alexa Fluor 555 (IF; 1:500)	Fisher	Cat#A-21432 RRID: AB_2535853
Donkey anti-Mouse IgG (H + L) Highly Cross-Adsorbed Secondary Antibody, Alexa Fluor 647 (IF; 1:500)	Fisher	Cat#A-21447 RRID: AB_141844
Donkey anti-Rat IgG (H + L) Highly Cross-Adsorbed Secondary Antibody, Alexa Fluor 594 (IF; 1:500)	Fisher	Cat#A-21209 RRID: AB_2535795
Donkey anti-Rabbit IgG (H + L) Highly Cross-Adsorbed, Alexa Fluor™ 350 (IF; 1:500)	Fisher	Cat#A-10039 RRID: AB_2534015
PE/Cyanine7 anti-mouse CD31 Antibody (Flow; 1:25)	BioLegend	Cat#102,418 RRID: AB_830757
GLAST (ACSA-1) Antibody, anti-human/mouse/rat (Flow; 1:100)	Miltenyi Biotec	Cat#130–118–483 RRID: AB_2733472
Recombinant APC Anti-VEGF Receptor 2 antibody (Flow; 1:100)	BioLegend	Cat# 136,405 RRID: AB_2044066
Oligodendrocyte Marker O1 Alexa Fluor® 700-conjugated Antibody (Flow; 1:100)	R&D systems	Cat# FAB1327N RRID: unknown
Oligodendrocyte Marker O4 Alexa Fluor® 700-conjugated Antibody (Flow; 1:100)	R&D systems	Cat# FAB1326N RRID: unknown
Alexa Fluor® 700 anti-mouse CD45 Antibody (Flow; 1:100)	BioLegend	Cat# 103,127 RRID: AB_493715
Alexa Fluor® 700 anti-mouse/human CD11b Antibody (Flow; 1:100)		Cat# 101,222 RRID: AB_493705
**Chemicals, peptides, and recombinant proteins**		
SU5416 (25 µM)	Sigma	Cat# S8442
SU1498 (2.5 µM)	Sigma	Cat# SML1193
VEGF nAb (10 µg/ml)	Genentech Inc	Pan et al., 2007
Mouse recombinant VEGF164 (10 ng/ml)	R&D Systems	Cat# AF493SP
Animal-Free Recombinant Human EGF, PeproTech (AF10015-500UG)	VWR	Cat#: 10,781–694
PeproTech Recombinant Human FGF-Basic (154A.A) (100-18B-250UG)	VWR	Cat#: 10,771–938
Lipofectamine 2000	Invitrogen	Cat#: 11,668–019
Gibco™ HBSS without Calcium, Magnesium or Phenol Red	Fisher	Cat#: 14–175-095
Gibco™ (Phosphate Buffered Saline) Solution, pH 7.4, 1 ×	Fisher	Cat#: 10–010–049
Albumin, Bovine Serum, Fraction V, Fatty Acid-Free, Nuclease- and Protease-Free	Millipore Sigma	Cat#: 126,609
TRITON™ X-100	Thermo Scientific Chemicals	Cat#: 21,568
5-Bromo-2′-deoxyuridine	Sigma	Cat# B5002
5-Ethynyl-2′-deoxyuridine	Click Chemistry Tool Kit	Cat#1324
TAPI-1	Fisher	Cat#61–621
Commercial assays/kits		
RNAscope Multiplex Fluorescent Assay v2	ACD Biotechne	Cat#: 323,136
RNAscope probe Mm-Vegfa-ver2	ACD Biotechne	Cat#: 412,261
RNAscope probe Mm-Kdr-C2	ACD Biotechne	Cat#: 414,811-C2
Mouse VEGF DuoSet ELISA	R&D Systems	Cat# DY493-05
Click-&-Go EdU 647 Cell Proliferation Assay Kit	Click Chemistry Tool Kit	Cat# 1329
**Deposited resources**		
CRISPRi backbone	Addgene	#196988
CRISPRi NT	Addgene	#196989
CRISPRi αVEGFR2	Addgene	#196990
**Cell lines**		
Cultured Neural Stem Cells	Babu et al., 2011	10.3389/fnins.2011.00089
bEnd.3 s	ATCC	Cat#2299 RRID:
HUVECs	Invitrogen	Cat# C0035C RRID:
**Mouse models**		
Wild-type C57BL/6 J	Jackson	#000664
VEGF^lox/lox^		Gerber et al., 1999
Nestin-GFP	Jackson	Jackson #033927
Lentiviral vectors		
mCherry	Kirby et al., 2015	
mCherry Cre	Kirby et al., 2015	Addgene no. 27546
GFP^+^ scramble shRNA	Mosher et al., 2012	
GFP^+^ *Vegfa* shRNA	Mosher et al., 2012	
**Software**		
ImageJ		https://imagej.nih.gov/ij/
Zen	Zeiss	
FlowJo 10.8.1	BD Life Sciences	
Image Studio	LI-COR	
Prism	GraphPad	https://www.graphpad.com/scientific-software/prism/

### Cell Culture Pharmacological Treatment of NSCs and HUVECs

SU5416 and SU1498 (Sigma) were dissolved in DMSO (10 mM) and stored at − 20 °C until use. SU5416 was used at 25 µM unless otherwise noted, and SU1498 was used at 2.5 µM. VEGF nAb in sterile PBS (B20-4.1.1) was provided by Genentech Inc. (Pan et al., 2007) and used at 10 µg/ml unless otherwise noted. Mouse recombinant VEGF164 (R&D Systems) was dissolved in sterile PBS and stored at − 20 °C. It was used at 10 ng/ml unless otherwise noted.

### Immunoblotting of Cultured NSCs and HUVECs

For p/AKT and p/Erk immunoblotting, NSCs were plated at a density of 300,000 cells/well in 6-well plates and treated at ~ 70% confluency. HUVECs were plated on uncoated 12-well plates at a density of 50,000 cells/well and maintained for 2 days, then serum-starved for 6 h before treatment. NSC and HUVEC monolayers were lysed with RIPA buffer (Pierce) with 1 × Halt Protease and Phosphatase Inhibitor (Thermo Scientific) on ice for 10 min. Scraped lysates were freeze-thawed three times and then centrifuged at 14,000 rpm for 10 min at 4 °C. The total protein concentration of the supernatant was quantified using a BCA kit (Pierce). Lysates were run on 4–12% bis–tris gels in 1 × NuPage MOPS buffer (Invitrogen) at 120 V for 2 h. Gels were transferred to a nitrocellulose membrane overnight in 20% methanol in NuPage Transfer Buffer (Invitrogen) at 4 °C. Membranes were blocked in 5% milk in 0.1 M TBS with 0.5% Tween20 (TBS-t) then incubated in primary antibody overnight at 4 °C in 5% milk in TBS-t. The secondary antibody was applied for 1 h at room temperature in 5% milk in TBS-t. Proteins were visualized and quantified on a LICOR Odyssey Clx infrared imaging system. Quantification was taken as the ratio of phosphorylated protein to its unphosphorylated form.

### Lentiviral Vector Production

mCherryCre (Addgene no. 27546) and mCherryonly vectors are described in [39]. Plasmids were packaged in vesicular stomatitis virus-glycoprotein G (VSV-G) lentivirus by the Cincinnati Children’s Hospital Viral Vector Core. VEGF and scramble shRNA lentiviral vectors are described in [45] and were packaged in VSV-G lentivirus by either Vigene Biosciences or the Stanford Gene Vector and Virus Core.

### Neighbor Rescue Experiment

Adult DG NSCs were grown in standard media on coated (short-term, forming a monolayer) or uncoated (long-term, forming spheres) tissue culture plates, allowing them to form spheres. On day 1, 5000 NSCs derived from DG of 6-week-old VEGF^lox/lox^ mice or VEGF^wt/wt^ mice were infected with mCherryCre or mCherryonly lentiviral vectors titered to result in < 5% total mCherry^+^ cells. In the short-term neighbor rescue experiment, 4 days after infection, NSC monolayers were incubated with 20 µM 5-bromo-2′-deoxyuridine (BrdU) (Sigma) for 2 h and then fixed with 4% paraformaldehyde prior to immunohistochemical processing. Long-term neighbor rescue NSC spheres were dissociated every 4 days, and freshly plated single cells were imaged live to quantify the percent of mCherry^+^ cells among those identified by brightfield contrast. Images were quantified by a blinded observer.

### VEGF ELISA of VEGF^lox/lox^ Cell Culture Conditioned Media

Adult DG VEGF^lox/lox^ NSCs were infected with mCherryCre or mCherryonly lentiviral vectors titered to result in < 5% mCherry^+^ cells (low) or > 50% mCherry^+^ cells (high). After 4 days, conditioned media was collected and centrifuged at 1000 g for 5 min. Conditioned media supernatant was extracted and processed with the Mouse VEGF DuoSet ELISA (R&D Systems) according to manufacturer instructions.

### shRNA Lentiviral Infection in Cultured NSCs and Scratch Assay

For confirmation of VEGF knockdown, adult Wt C57BL/6J mouse DG-derived NSCs were plated on coated 96-well plates at a density of 10,000 cells/well, then infected with lentiviral vectors containing a *Vegfa* shRNA or a scramble shRNA (described in [45]) at a MOI of 50. Media was changed after 24 h. After 4 days, conditioned media was collected from the supernatant and assayed with the Mouse VEGF DuoSet ELISA (R&D Systems) according to manufacturer instructions.

For the scratch assay, NSCs were plated into a 24-well plate coated with laminin and PDL at a density of 20,000 cells per well. Twenty-four hours later, cells were treated with *Vegfa* or scramble virus. After 72 h, a scratch was performed as above and imaged 1 h and 8 h afterwards to track NSC migration. At 1 h, the distance from the scratch edge to the center was taken as baseline within each well. At 8 h, the distance of individual GFP^+^ and GFP^−^ cells (*n* =  ~ 30 cells each/experiment) was measured using Zen software. Because each scratch was unique, the distance to midline was expressed as a change relative to the initial distance from edge to midline, such that a cell remaining at the edge of the scratch would get a 0 and a cell reaching the midline would get a 1.

### Stereotaxic Surgery

The mice that received stereotaxic infusion of lentiviral vectors were also reported on in [12]. We reproduce those methods here for convenience: “Mice were anesthetized by inhalation of isoflurane (Akorn, 5% induction, 1–2% maintenance) in oxygen and mounted in the stereotaxic apparatus (Stoelting). Ocular lubricant (Puralube) was placed over the eyes to prevent evaporative dry eye. Following sterilization with alcohol (Fisher) and betadine swabs (Fisher), the skull was exposed and the lambda and bregma sutures were aligned in the same horizontal plane. A small bur hole was drilled in the skull and an automated injector (Stoelting) with a Hamilton syringe (Hamilton) was lowered to the injection depth at a rate of −1.0 mm/min. Mice were injected with 0.5 μL of scramble control shRNA virus into one hemisphere and 0.5 μL of Vegfa shRNA virus into the contralateral hemisphere at a rate of 0.1 μL/min. The injection coordinates from bregma were: anterior/posterior −1.9 mm, medial/lateral ± 1.6 mm, −1.9 mm dorsal/ventral from dura. Post-surgery, the incision was sealed with tissue adhesive (3 M) and the mouse was given saline (Hospira) and carprofen (Zoetis) injection i.p. After 21d, mice were perfused for immunohistochemical processing.”

### Immunohistochemical Processing and Imaging

#### Cultured NSCs

Adherent NSCs were fixed with 4% paraformaldehyde, then rinsed with PBS three times before incubating in a blocking solution containing 1% normal donkey serum (Jackson ImmunoResearch) and 0.3% Triton X-100 (Acros) in PBS. Cells were then incubated in primary antibody diluted in blocking solution overnight at 4 °C. The following day, after three rinses in PBS, cells were incubated in secondary antibodies diluted in blocking solution for 2 h. If a biotinylated secondary was used, a fluorophore-conjugated tertiary was applied for 1 h diluted in PBS before rinsing and nuclei counterstaining with Hoechst (10 min, 1:2000 in PBS) (Invitrogen). If proceeding for BrdU labeling, the cells were rinsed and fixed with 4% paraformaldehyde in 0.1 M PB for 10 min, rinsed with PBS three times and incubated with 2N HCl for 30 min at 37 °C. After three PBS rinses and 30 min incubation in blocking, cells were incubated in BrdU primary antibody diluted in blocking solution overnight at 4 °C. The next day, cells were rinsed three times with PBS and exposed to a secondary antibody diluted in blocking solution for 2 h before Hoechst counterlabeling. Cells in 96-well plates were imaged immersed in PBS using × 10 magnification, while those on chamber slides were coverslipped with Prolong Gold Antifade Mountant (Fisher) and imaged using × 40 oil magnification, both with a Zeiss apotome digital imaging system (Zeiss).

#### Brain Sections

The same procedures as [12] were followed, which we reproduce here for convenience: “Brains for immunolabeling were harvested following perfusion with ice-cold PBS followed by fixation in 4% paraformaldehyde overnight at 4 ˚C. After equilibration in 30% sucrose in PBS, 40 μm coronal brain sections were obtained in 1 in 12 series on a freezing microtome (Leica) and stored in cryoprotectant at −20 ˚C. Sections were rinsed 3 × in PBS and incubated in a blocking solution containing 1% normal donkey serum and 0.3% Triton X-100 (Acros) in PBS before incubation in primary antibodies for 24–72 h. Sections were rinsed 3 × with PBS and exposed to a secondary antibody diluted in blocking solution for 2 h. For VEGF-GFP co-labeling with nestin and VEGFR2, sections were incubated with a biotinylated secondary for GFP labeling followed by a streptavidin alexafluor 488 tertirary (1:1000 in PBS). Sections were then rinsed, blocked and incubated in anti-BrdU primary and appropriate secondaries as above. The DG of the hippocampus was imaged in 1 μm Z-stacks at 20 × magnification using a Zeiss apotome digital imaging system (Zeiss).”

### Immunofluorescent Image Quantification

In mice infused with shRNA lentiviral vectors, NSPCs were identified. RGL-NSCs were identified by GFAP^+^/SOX2^+^ colocalization and GFAP^+^ radial processes extending from the SGZ towards the inner molecular layer, while GFAP^−^/SOX2^+^ cells in the SGZ layer were identified as IPCs and then categorized as GFP^+^ or GFP^−^ within the SGZ, yielding a percent of GFP^+^ or GFP^−^ NSPCs that were RGL-NSC or IPC phenotype. The SGZ was defined as the zone spanning two cell body widths between the dense granular cell layer and the hilus. Cell counts were performed manually in Zen by a blind observer and corrected for the area of SGZ sampled, yielding a density. Cell distance to vessels was quantified as described in [12]: “Endothelia were identified by CD31^+^. Distance to vasculature was measured as the distance from the middle of a cell body to the nearest CD31^+^ vessel. Random distances for vessel associations were measured by sampling the distance of random Hoechst^+^ cells in the middle of the SGZ to the vasculature.” In VEGF-GFP mice, *n* = 4 mice were immunolabeled and VEGFR2/VEGF-GFP co-localization with Nestin was qualitatively assessed and found to be abundant throughout the DG of all mice.

### CRISPRi VEGFR2 Knockdown for Immunolabeling

bEnd.3 cells or NSCs were plated on uncoated (bEnd.3) or on PDL/laminin-coated (NSCs) glass eight-chamber slides (Fisher) and allowed to adhere for 24 h. After 24 h, cells were transfected with CRISPRi expressing plasmids using Lipofectamine 2000 (Fisher), according to manufacturer instructions. Media were changed to remove lipofectamine/DNA 5 h later and replaced with standard growth media for each respective cell type. Two days later, cells were fixed with 4% paraformaldehyde.

### Plasmid Construction and sgRNA Design

The CRISPR interference (CRISPRi) lentivirus construct was modified from pLV hU6-sgRNA hUbC-dCas9-KRAB-T2a-GFP (Addgene #71,237, a gift from Charles Gersbach, [46]), which expresses all necessary CRISPRi machinery (both the dCas9-KRAB and sgRNA) from the same plasmid. The UbC promoter was replaced with a Nestin regulatory element comprised of the Nestin promoter and second intronic enhancer from [47] to create pLV hU6-sgRNA Nestin-dCas9-KRAB-T2a-GFP. Additionally, two Esp3I recognition sites were placed after the U6 promoter for restriction cloning of sgRNA insert sequences. The insert sequences were synthesized as single strand oligonucleotides (Integrated DNA Technologies) in the form of 5′ GGACG(N)20 3′ and 5′ AAAC(N′)20C 3′ where (N)20 refers to the sequence of the sgRNA and (N′)20 is the reverse complement. These oligonucleotides were annealed together and ligated with Esp3I-digested pLV hU6-sgRNA Nestin-dCas9-KRAB-T2a-GFP to obtain the final constructs for lentivirus packaging. The sgRNAs targeting the region − 50 to + 300 bp relative to the transcriptional start sequence of *Kdr* were designed using the CRISPRi function of the Broad Institute GPP portal (https://portals.broadinstitute.org/gpp/public/analysis-tools/sgrna-design). The top 5 ranked sgRNAs returned by the GPP tool were chosen and a non-targeting sgRNA [48] was used as control.

### VEGFR2 Cell Surface and Intracellular Immunolabeling and Quantification

To label all VEGFR2, the blocking solution was 1% normal donkey serum and 0.3% Triton X-100 in PBS. To label only cell surface VEGFR2, Triton X-100 was omitted but all other procedures were identical. Three random view fields were captured for VEGFR2 intensity quantification using thresholded area and intensity within cell bodies using ImageJ.

### TAPI-1 Treatment

NSCs derived from 6-week-old Wt C57BL/6J DG were plated at 20,000 cells/well on PDL/laminin-coated glass eight-chamber slides and allowed to adhere for 24 h. TAPI-1 (Fisher) was added to media at 0–80 µg/ml. NSCs were fixed 24 h later. For TAPI rescue experiments, DG NSCs were treated with 160 µg/ml 24 h before 10 min of VEGF164 treatment (10 ng/ml, R&D Systems).

### CRISPRi VEGFR2 Knockdown for qPCR

bEnd.3 cells were plated on 12-well tissue culture plates and allowed to grow to 80% confluency before transfection with CRISPRi plasmids using Lipofectamine 2000 (Fisher), according to manufacturer instructions. Eighteen hours later, cells were harvested by scraping in 0.25% Trypsin–EDTA with phenol red (Fisher), then centrifuged 1000 g for 5 min. RNA was isolated from cell pellets using the Aurum Total RNA Mini kit (Bio-Rad). Total RNA was quantified using a BioTek Epoch Microplate Spectrophotometer, and 250 ng RNA was converted to cDNA using the iScript cDNA synthesis kit (Bio-Rad) and a ThermoFisher Applied Biosystems 2720 Thermal Cycler. cDNA was amplified with gene-specific primers and SsoAdvanced Universal SYBR Green Supermix in a Bio Rad CFX96 Touch Real-Time PCR Detection System. Melt curves were used to confirm the purity of the amplified product, and the Ct value was normalized to housekeeping gene Hprt. ΔΔCt values were used to obtain fold change over non-target control average.

Hprt: PrimerBank ID: 96975137c1.

Forward primer: agtcccagcgtcgtgattag.

Reverse primer: tttccaaatcctcggcataatga.

Kdr: From [39]

Forward primer: atctttggtggaagccacag.

Reverse primer: ccatgatggtgagttcatcg.

### Diagram Creation

All cartoons in this manuscript were created with BioRender.com.

### Quantification and Statistical Analysis

Statistical test details are in the statistical reporting spreadsheet. Western blots for Akt and Erk phosphorylation after SU5416, VEGF nAb, or recombinant VEGF treatment were compared to control using one-way ANOVA with Dunnett’s multiple comparison test. Two-way ANOVAs were used to compare the effect of mCherryCre and mCherryonly in the neighbor rescue experiment with Tukey’s or Sidak’s multiple comparison post hoc tests. Three-way ANOVAs were used to compare virus × cell type × GFP expression interaction in viral infected tissue sections, followed by Sidak’s multiple comparisons between groups differing by 1 factor. The effect of TAPI-1 on VEGFR2 immunoreactivity was compared to control using one-way ANOVA with Kruskal–Wallis multiple comparison test. TAPI rescue of VEGF signaling through pAkt expression was analyzed by two-way ANOVA with Sidak’s multiple comparison post hoc tests. All analyses were performed using Prism (v9.0 or above; GraphPad Software) and *p* < 0.05 was considered significant.

Supplementary Information.

## Supplementary Information

Below is the link to the electronic supplementary material.

Supplementary file1 (DOCX 1238 KB)

Supplementary file2 (XLSX 21.1 KB)

## Data Availability

Data is provided within the manuscript, statistics table or supplementary information files.
