# Supplementary material for: Intracrine VEGF Signaling Is Required for Adult Hippocampal Neural Stem Cell Maintenance and Vascular Proximity
Source: Mol Neurobiol. 2025 Mar 25;62(8):9604–24. doi: 10.1007/s12035-025-04861-1 (PMC12289830; doi:10.1007/s12035-025-04861-1)

**Supplemental Information**

**
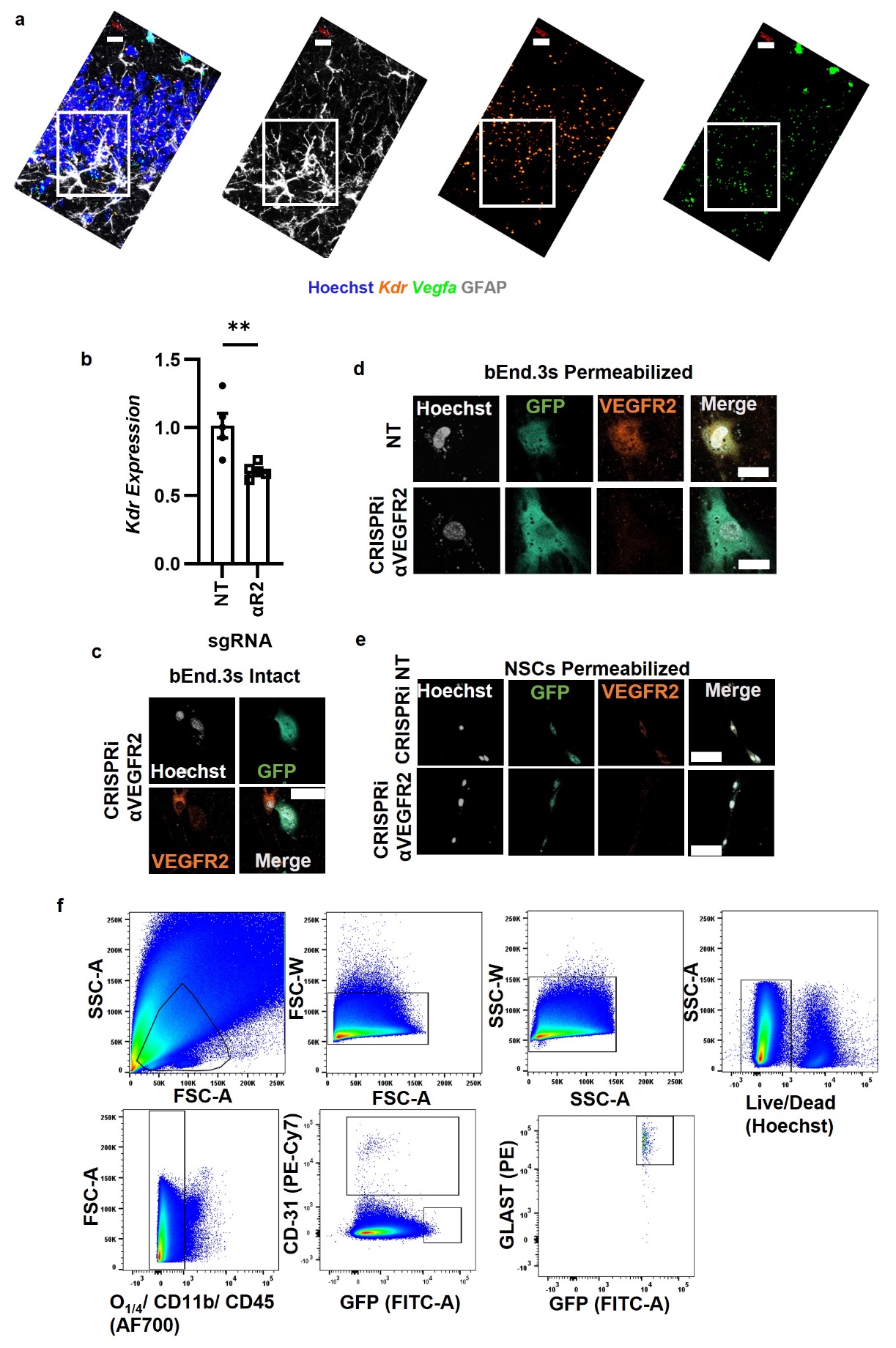
Fig. S1 NSCs lack immunoreactive VEGFR2 on the cell surface.** a) Wider-field of view of image from Fig 1A. White box shows area from Fig 1A. Scale = 10 µm. Kdr mRNA expression in bEnd3.s after transfection with plasmids expressing an sgRNA targeting VEGFR2. N = 3 wells/grp/exp, 1 exp; mean ± SEM. c,d) Representative images of VEGFR2 immunoreactivity in intact (c) or permeabilized (d) cultured bEnd.3s following transfection with CRISPRi NT or αVEGFR2. e) Representative images of VEGFR2 immunoreactivity in permeabilized cultured NSCs following infection with CRISPRi NT or αVEGFR2. f) Representative flow cytometry profiles for endothelial and NSC subsets


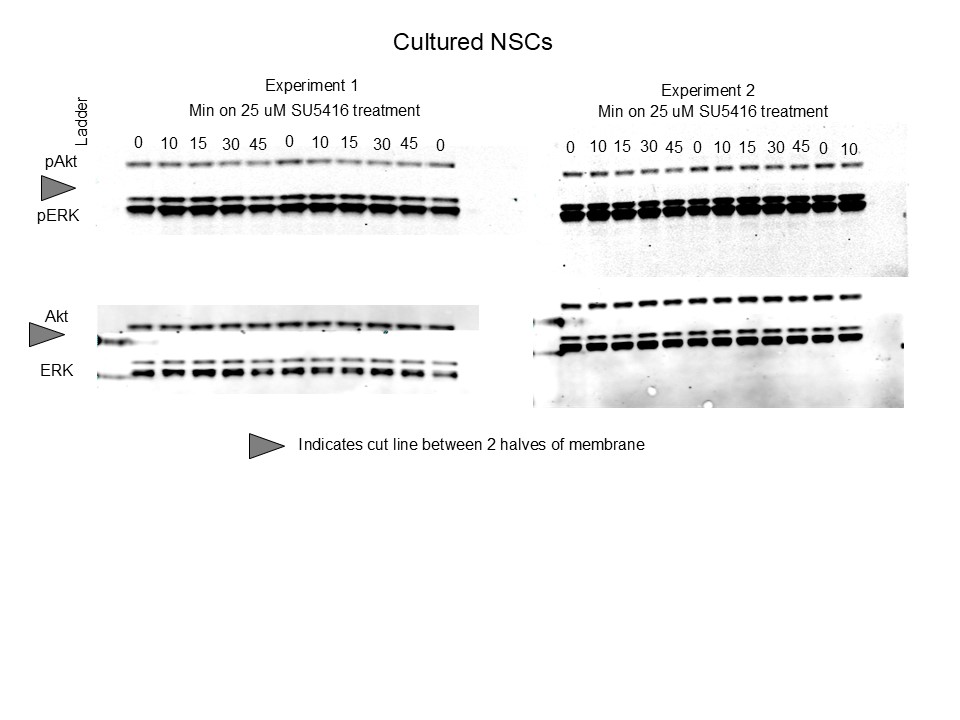

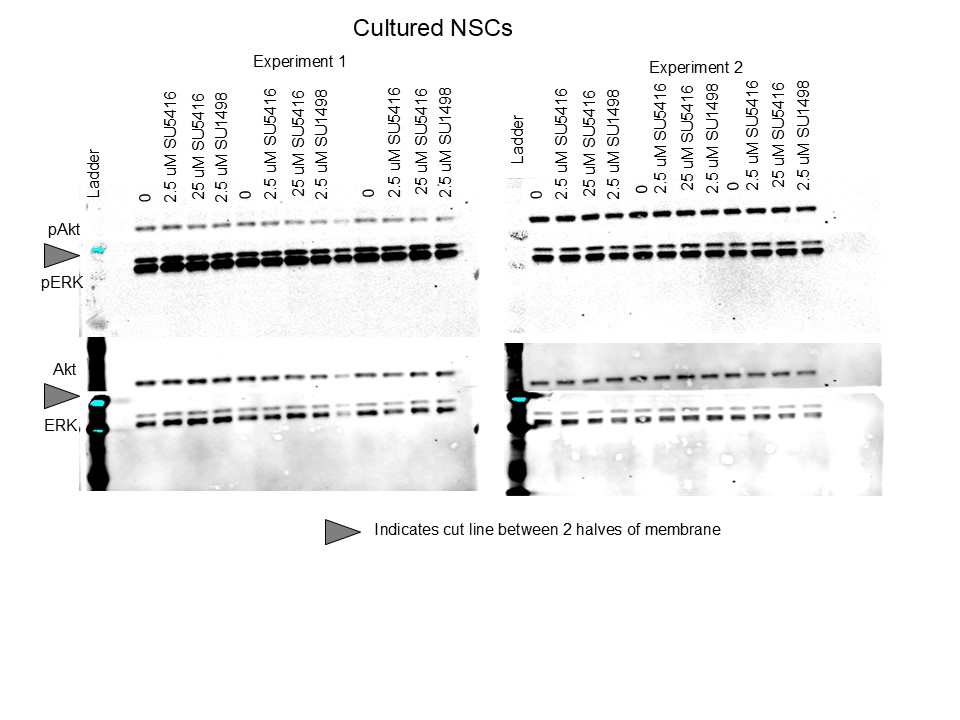
**Fig. S2: Raw western blots.** Each set of images includes labels with treatments and cell type being assayed. Membranes were physically trimmed prior to imaging to image only weight ranges of interest. ERK images are included because they are part of the raw, unaltered image file. ERK data was not presented or used in the main manuscript.


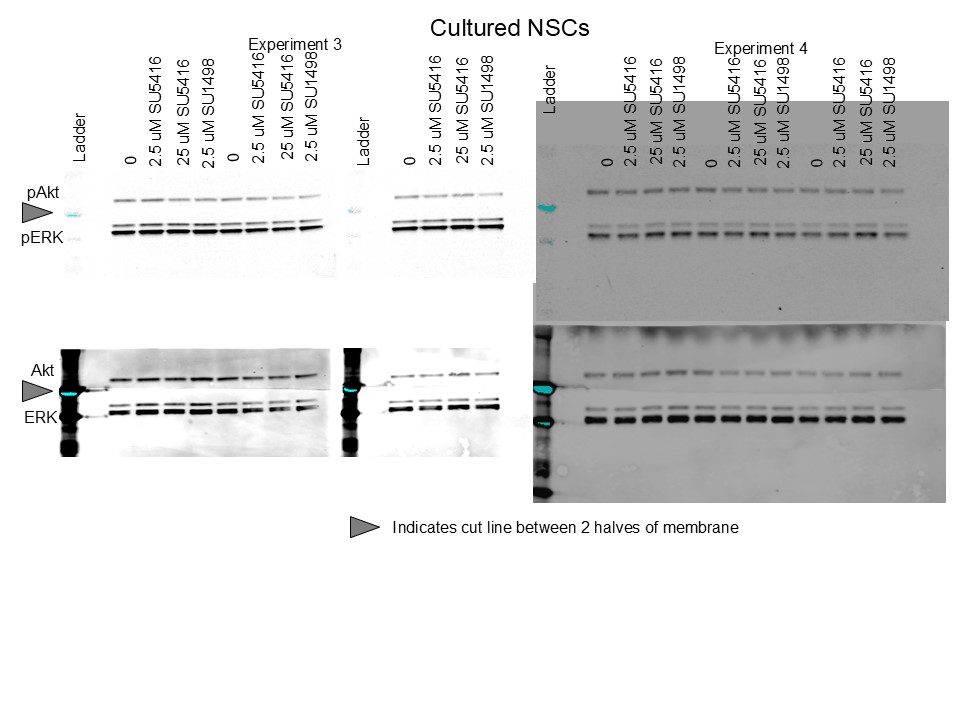

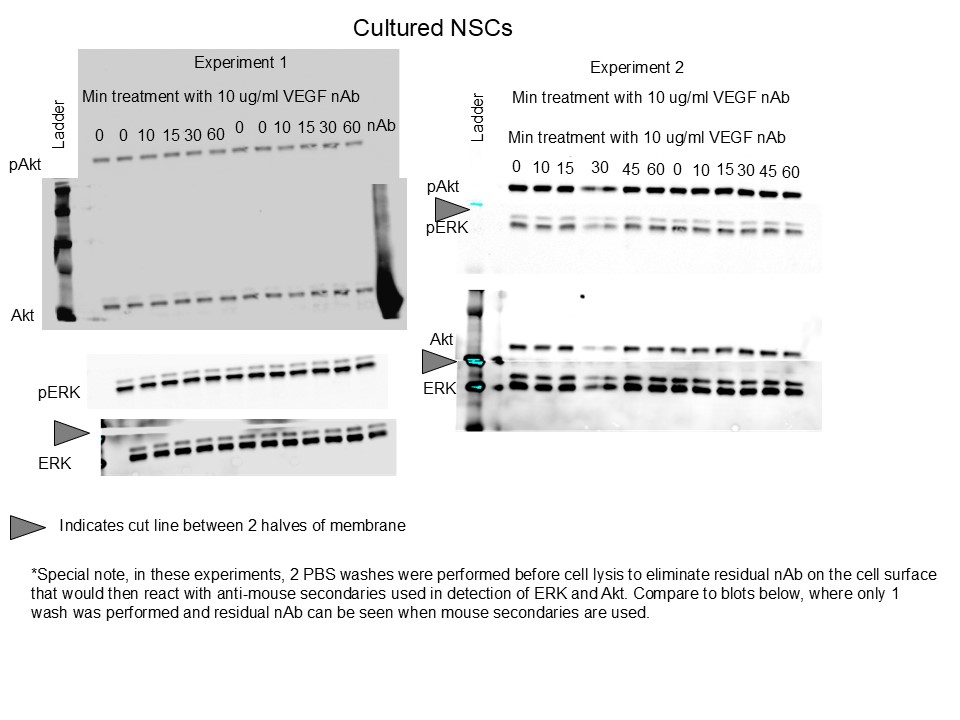

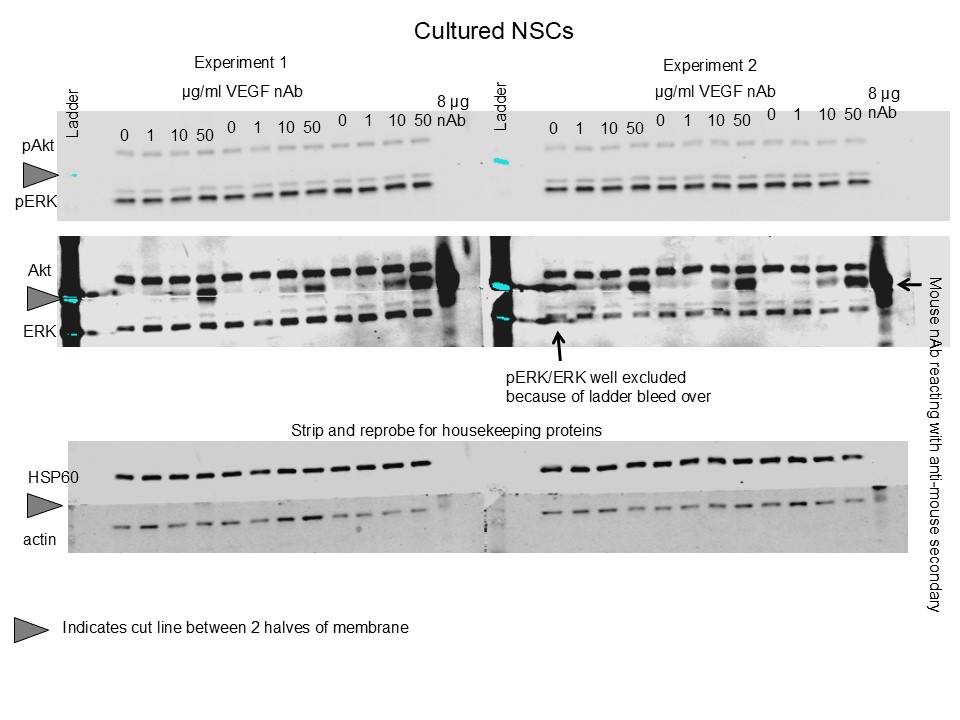

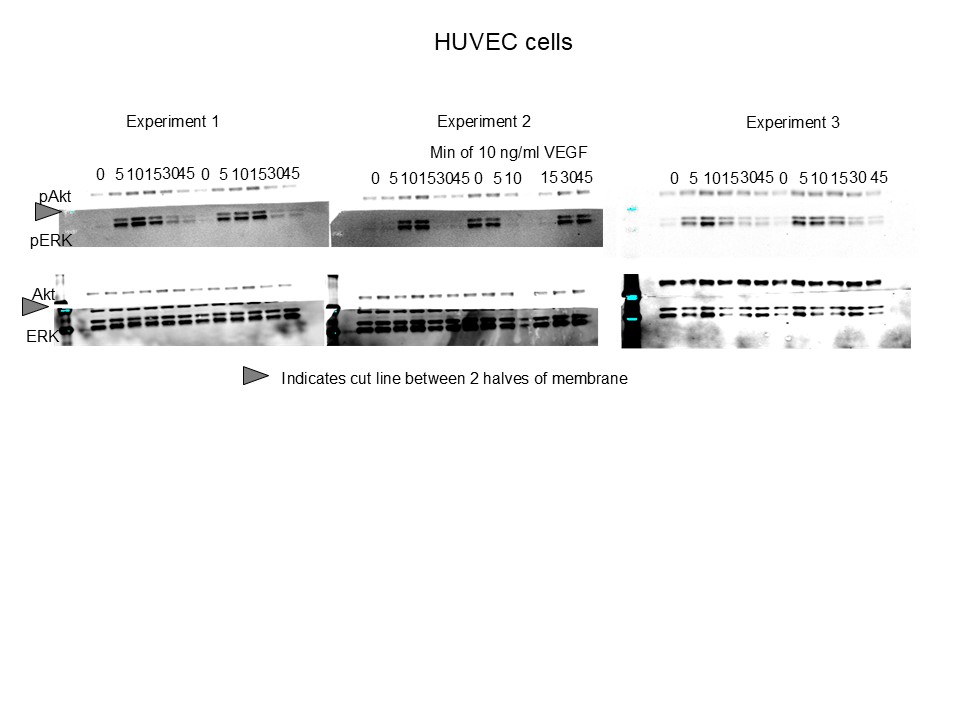

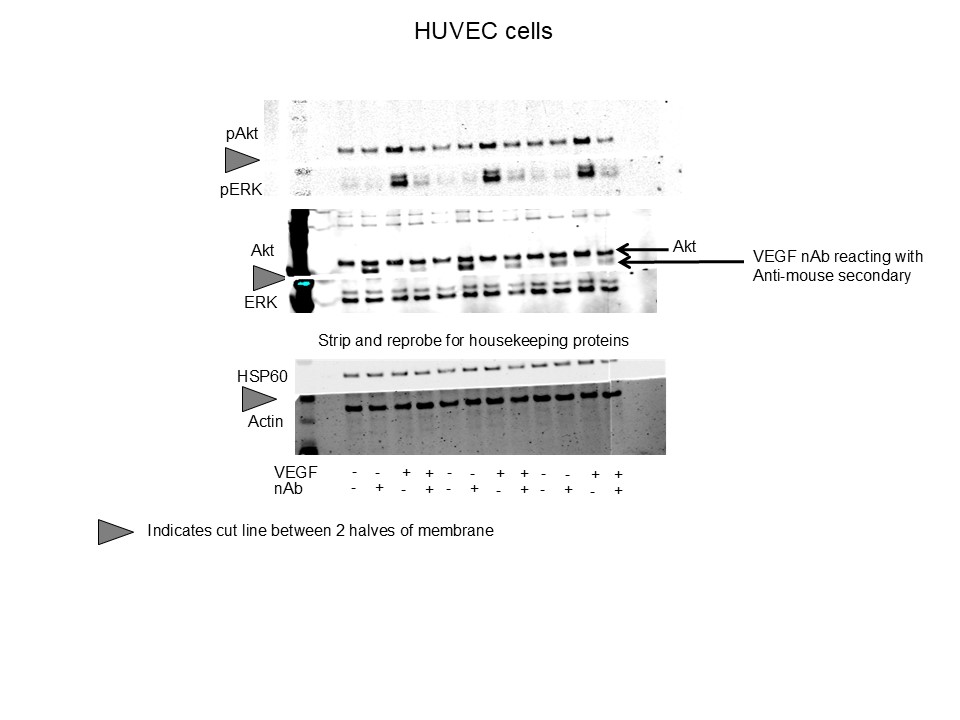

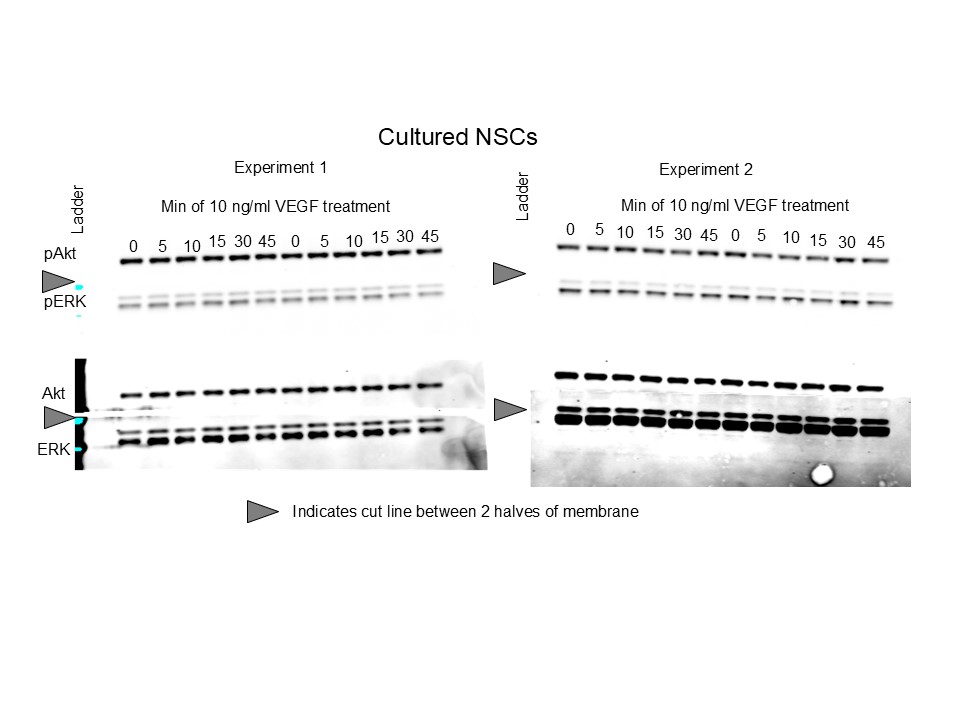

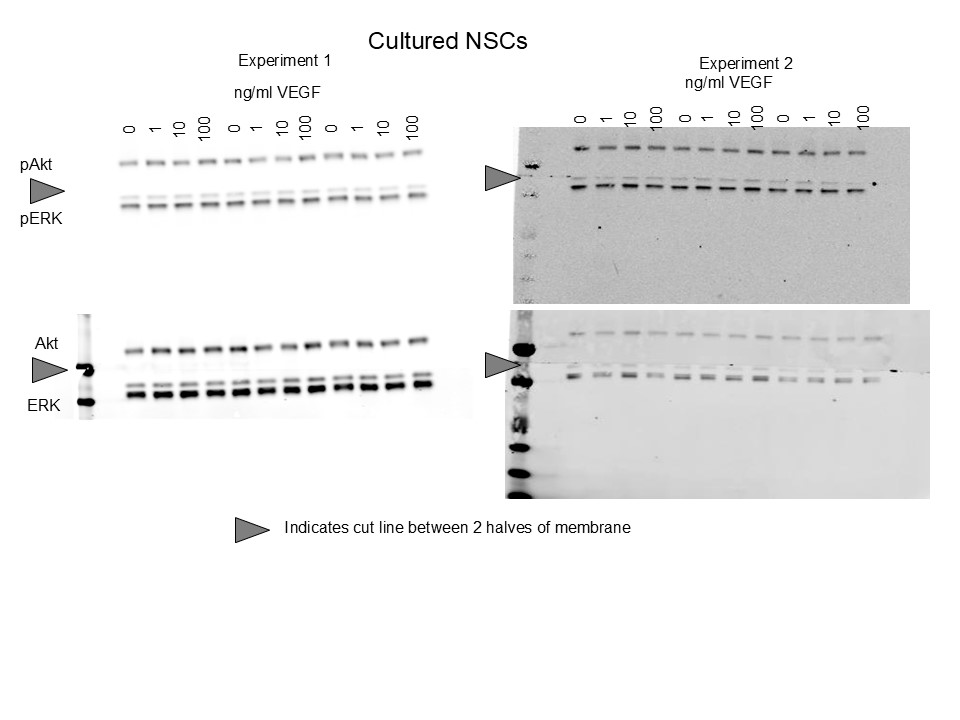

Supplement: Supplementary file 1 — Supplementary file1 (DOCX 1238 KB) [file 12035_2025_4861_MOESM1_ESM.docx]
